# Rapidly Evolving Genes and Stress Adaptation of Two Desert Poplars, *Populus euphratica* and *P. pruinosa*

**DOI:** 10.1371/journal.pone.0066370

**Published:** 2013-06-11

**Authors:** Jian Zhang, Penghui Xie, Martin Lascoux, Thomas R. Meagher, Jianquan Liu

**Affiliations:** 1 Molecular Ecology Group, State Key Laboratory of Grassland Agro-ecosystem, School of Life Sciences, Lanzhou University, Lanzhou, Gansu, China; 2 Laboratory of Evolutionary Genomics, CAS-MPG Partner Institute for Computational Biology, Chinese Academy of Sciences, Shanghai, China; 3 Program in Evolutionary Functional Genomics, Evolutionary Biology Centre, Uppsala University, Norbyvägen, Uppsala, Sweden; 4 School of Biology, University of St Andrews, St Andrews, United Kingdom; CNR, Italy

## Abstract

Understanding which genes have evolved rapidly with the recent tree speciation in arid habitats can provide valuable insights into different adaptation mechanisms. We employed a comparative evolutionary analysis of expressed sequence tags (ESTs) from two desert poplars, *Populus pruinosa* and *P. euphratica*, which diverged in the recent past. Following an approach taken previously with *P. euphratica*, we conducted a deep transcriptomic analysis of *P. pruinosa*. To maximize representation of conditional transcripts, mRNA was obtained from living tissues of two types of callus and desert-grown trees. *De novo* assembly generated 114,866 high-quality unique sequences using Solexa sequence data. Following assembly we were able to identify, with high confidence, 2859 orthologous sequence pairs between the two species. Based on the ratio of nonsynonymous (*Ka*) to synonymous (*Ks*) substitutions, we identified a total of 84 (2.9%) ortholog pairs exhibiting rapid evolution with signs of strong selection (*Ka/Ks*>1). Genes homologous to these ortholog pairs in model species are mainly involved in ‘responses to stress’, ‘ubiquitin-dependent protein catabolic processes’, and ‘biological regulation’. Finally, we examined the expression patterns of candidate genes with rapid evolution in response to salt stress. Only one pair of orthologs up-regulated their expression in both species while three and four genes were found to up-regulated in *P. pruinosa* and in *P. euphratica* respectively. Our findings together suggest that the genes at the same category or network but with differentiated expressions or functions may have evolved rapidly during adaptive divergence of the two species to differentiated salty desert habitats.

## Introduction

Rapidly evolved genes with a higher divergence than the remaining pairs of genes during recent speciation of closely related species are assumed to be responsible for differentiated habitat adaptation [1]–[3]. Some of these genes may be companied by changed spatial and temporal expressions [4]–[5] possibly due to their critical mutations [5]. In addition, these mutations, if advantageous and fixed within population(s), should be favoured by natural selection at the same time [6]. The rapidly evolved genes can be identified through assessment of nonsynonymous (*Ka*) and synonymous (*Ks*) substitution rates between orthologous genes with protein-coding functions [7]–[10]. Paired genes with *Ka/Ks*>1 are inferred to have experienced rapid evolutions favoured by natural selection [11], [12]. Detection of such effects in non-model organisms has been limited by the availability and cost of sequence data. ‘Next generation’ sequencing provides an opportunity to obtain abundant and inexpensive genomic data for non-model species, especially transcriptome sequences [10], [13]. Such sequences are being used to detect genes showing rapid evolution in the recent past across the entire genomes [12], [14]–[17].

In this study, we used next-generation high-throughput transcriptomic sequencing (Illumina Hiseq 2000 *De novo* transcriptome sequencing) to detect genes under positive selection between two poplar species in ecologically divergent salty deserts: *Populus euphratica* and *P. pruinosa*. These two species comprise a monophyletic lineage as a separate section (sect. *Turanga*) [18]–[20]. *P. euphratica* has been used as a model species for studying abiotic responses to salt or drought stress [21]–[24]. In addition to morphological differences between the two species in leaves and hairs, they also occur in different habitats. Both species occur in deserts with high summer temperatures [18]. However, *P. euphratica* occurs in drier deserts with low levels of underground salty water while *P. pruinosa* occurs in less dry deserts with higher salt levels and higher levels of underground water. These two species are likely to have diverged due to ecological separation in spite of ongoing gene flow [20]. Genes undergoing rapid evolution during this recent speciation may be involved in differential habitat adaptation.

Transcriptome sequences of *P. euphratica* were recently reported [24]. Building upon this earlier work, our objectives in the present study were (1) to present a *de novo* assembly of the *P. pruinosa* transcriptome using Solexa data and obtain orthologous gene pairs from transcriptomic datasets of *P. euphratica* and *P. pruinosa*, (2) to identify genes with rapid evolution indicating signs of positive selection, and (3) to examine whether expression changes of rapidly evolved genes are consistent or inconsistent with response to salt stress across two species. Our data were collected by sequencing cDNA libraries of living tissues from mature trees of two species growing in Tarim Basin desert, which had a long period to adapt to the local conditions before salt-stressed callus and unstressed callus were sampled. The identified genes will be of particular interest for physiological and molecular studies because they are sensitive to environmental stress experienced by these two species in deserts. We further predicted that such genes should have different expression patterns under salt stress because these two species are likely to have differentiated along different physiological pathways during interactions with salt and other stresses.

## Materials and Methods

### Plant Material

Following the approach used previously for *P. euphratica* [24], transcripome analyses of *P. pruinosa* were based on three sets of samples representing control callus, salt-stressed callus and desert-grown trees. For the desert-grown tree samples, we collected three replicate samples of roots, leaves, flower buds, flowers, xylem and phloem from two males and one mature female in the Tarim Basin desert in Xinjiang. Despite the fact that *P. pruinosa* always occur together with *P. euphratica* in their natural distributions, three collected *P. pruinosa* trees used for the present analyses occur far away from the nearby *P. euphratica*. These trees in the present study were selected in the same physiological state and age class as those used for *P. euphratica* [24]. No specific permits were required for the described field studies. In addition, we cultured *P. pruinosa* calli using the method described by Zhang et al. [25] under the same conditions with *P. euphratica*, replaced the growth medium of one set with fresh, unamended medium and that of another set with fresh medium supplemented with 100 mM NaCl (to impose salt stress), and then harvested both sets 24 h later. The callus from *P. euphratica* and *P. pruinosa* has the same passage number and they were highly comparable in terms of origin, physiological state, age, cell types present, morphology, and growth rate within the experimental error. We stored all samples at −80°C prior to RNA extraction.

### RNA Extraction and Quality Determination

Total RNA was extracted three times from each of the sample sets, using a CTAB procedure [26]. *A*260/*A*280 ratios of the RNA samples dissolved in 10 mM Tris (pH 7.6) ranged from 1.9 to 2.1. The integrity of the RNA samples was examined with an Agilent 2100 Bioanalyzer and their RIN (RNA integrity number) values ranged from 8.6 to 10.0, with no sign of degradation. RNA from each replicate was pooled (in equal volumes) to obtain a single RNA sample for cDNA preparation and RNA-Seq, and equal amounts of mRNA from different tissues of the desert-grown trees were pooled to make single samples.

### cDNA Library Construction and Illumina Sequencing

cDNA library construction and sequencing were performed by BGI (Shenzhen, China). The entire process followed a standardized procedure and monitored by BGI's Quality Control System. For cDNA synthesis and Solexa sequencing, 20 µg of total RNA was used, at a concentration of ≥400 ng/µl. Poly(A) mRNA was first isolated using oligo dT beads. Then, mRNA was fragmented into small pieces using divalent cations for 5 min at an elevated temperature (70°C) according to the manufacturer's instructions (Illumina). Based on these cleaved RNA fragments, we used random hexamer-primer and reverse transcriptase (Invitrogen) to synthesize first-strand cDNA. Second-strand cDNA was synthesized using RNase H (Invitrogen) and DNA polymerase I (New England BioLabs). We constructed three paired-end cDNA libraries with insert sizes of 200 base pair (bp), and then sequenced the cDNA using an Illumina (San Diego, CA, USA) Genome Analyzer platform according to the manufacturer's protocols with a read length of 75 bp in two lanes. Image output data from the sequencer was transformed into raw sequence data by base calling.

### *De Novo* Assembly of the *P. Pruinosa* Transcriptome

We first cleaned raw sequence reads by removing exact duplicates from both sequencing directions. We further cleaned reads by removing adapter sequences as well as reads with too many (>8) unknown base calls (N), low complexity, and low-quality bases (>50% of the bases with a quality score ≤5). Cleaned reads from each library were assembled separately.

*De novo* assembly of the clean reads was performed using SOAPdenovo software [27] (http://soap.genomics.org.cn), which applies de Bruijn graph algorithm and a stepwise strategy. Briefly, the clean reads were firstly split into smaller pieces, the ‘k-mers’, for assembly to produce contigs using the de Bruijn graph. Next, the reads were realigned to the contig sequences, and the paired-end relationships between the reads were used to construct scaffolds between contigs. Then gap fillings were carried out using pair-end information to retrieve read pairs with one read well aligned on the contigs and another read located in the gap region. The resulting scaffolds with the least Ns were defined as unigenes. The unigenes assembled by short reads from three samples were further clustered into a non-redundant all-unigene set in a comprehensive transcriptome using the TGI Clustering Tool (TGICL) [28] with 50 bp overlap and a minimum of 90% identity.

To obtain high-quality sequences for further annotation and analysis, we excluded unigene sequences that might represent non-coding RNAs. Unigene sequences assigned to known non-coding RNAs (Rfam database: http://www.sanger.ac.uk/Software/Rfam/, release 10.0), microbial (MBGD: http://mbgd.genome.ad.jp/), fungal and virus (based on data downloaded from the NCBI database, fungal: http://www.ncbi.nlm.nih.gov/genome/?term=fungi; virus: http://www.ncbi.nlm.nih.gov/genome/?term=virus) sources were filtered out.

### Transcriptome Functional Annotation and Open Reading Frame Identification

We annotated the all-unigenes based on sequential BLAST searches [29] designed to find the most descriptive annotation for each sequence [24]. The assembled unique transcripts were determined by BLASTX against the NCBI non-redundant (Nr) protein database, the Swiss-Prot protein database (http://www.expasy.ch/sprot), the Kyoto Encyclopedia of Genes and Genomes (KEGG) pathways database [30], and the Cluster of Orthologous Groups (COG) database (http://www.ncbi.nlm.nih.gov/COG/), applying a E-value cutoff level of <1E − 5. Incongruent results from different databases were settled under a priority order of Nr, Swiss-Prot, KEGG, and COG. Proteins with the highest sequence similarity in blast results were taken to determine the open reading frames (ORF) with the given unigenes.

Based on their Nr annotations, the all-unigenes were assigned GO annotations using the Blast2GO program [31], followed by functional classification using the WEGO software [32], to identify the distribution of gene functions of the species from the macro level (the third GO level). Gene Ontology (GO: http://www.geneontology.org/) is an international standardized gene functional classification system which offers a dynamically updated controlled vocabulary and a strictly defined concept to comprehensively describe properties of genes and their products in any organism. Three ontologies, molecular function, cellular component and biological process, are provided in GO database.

### Identification of Rapidly Evolved Genes

Identification of orthologous ESTs between *P. euphratica* and *P. pruinosa* was performed using the bidirectional best hit (BBH) method [33]. This method outperforms more complex orthology identification algorithms [34]. Reciprocal batch BLASTP searches were carried out setting the expected value cut off to 1E − 5 to minimize significant matches to paralogous sequences. Hits with a bit score >400 were retrieved for further analysis. Based on translated proteins of the orthologous sequences, ESTs were aligned by the Probabilistic Alignment Kit (PRANK) software [35].

Due to the frequent genome duplications, it is difficult to distinguishing orthologs from paralogs in certain situations when transcriptome sequences are analyzed. In order to establish orthologous relationships between the paired of sequences, we used a series of strict criteria to exclude paralogs. These filtering methods included (i) only those with the corresponding best hits of two ESTs that had a length overlap >150 bp were retrieved; (ii) orthologous pairs with a percent coverage of 90% were retrieved; (iii) aligned sequences with unexpected stop-codons and ambiguous alignments were excluded from further analysis; (iv) all candidate orthologs with a synonymous (*Ks*) substitution value greater than 0.1 were excluded due to the possibility of being paralogs [1], [12]; and (v) all alignments were manually checked.

Once the putative ortholog pairs were aligned, nonsynonymous (*Ka*) and synonymous (*Ks*) substitution ratios were calculated between orthologous coding regions in KaKs_Calculator v1.2 [36] by using the maximum-likelihood YN model [37]. We calculated synonymous and nonsynonymous sites, synonymous and nonsynonymous substitutions, GC contents, and the sequence length after removing gaps and stop codons, in addition to synonymous and nonsynonymous substitution rates and their ratio. Meanwhile, the Fisher's exact test for small sample was applied to justify the validity of *Ka* and *Ks* values calculated by this method. To determine if there was any enrichment of GO categories in the positively selected group versus the non-selected group of orthologs, Fisher's exact test was also used to test for over-represented functional categories among positively selected genes (PSGs). For each category C and set of PSGs S, a 2×2 contingency table was constructed for the numbers of genes assigned or not assigned to C and within or outside S. Then, (one-sided) P values for the independence of rows and columns were computed by Fisher's exact test.

We randomly selected 10 pairs of ESTs showing apparent adaptive evolution (*Ka/Ks* >1) to examine further whether they are orthologs rather than paralogs. We designed common primers for each of these pairs of ESTs. We then used these primers to amplify, clone and sequence DNA from five other individuals of *P. euphratica* and *P. pruinosa*. If paired ESTs from two species were paralogs, DNA sequences amplified and sequenced according the conserved region of each pair of ESTs should comprise different gene copies in the same species. These DNA sequences should cluster into at least two groups and each group should include sequences from different species.

### Gene Expression MeasurEment

Gene expression levels were measured in RNA-Seq analyses as numbers of reads per kilobase per million mapped reads (RPKM) on exon regions within a given gene [38]. For a given all-unigene, two RPKM values were generated by mapping the subtranscriptome reads of the control and salt-stressed callus to it using SOAP2 with a maximum of three mismatches [39].

## Results

### *De Novo* Assembly of the Transcriptome of *P. Pruinosa*

After removing low-quality sequences and trimming adapter sequences, 28 million 75-bp paired-end clean reads were generated from each of the control-callus, salt-stressed callus and desert-grown tree cDNA libraries in the Illumina Genome Analyzer runs (Table S1). The percentage of Q20 bases for the clean reads in the three sub-transcriptomes were more than 95% (Table S1). In total, these clean reads constitute ∼12 GB of sequence data. The raw reads produced in this study have been deposited in the Short Read Archive (SRA) at the NCBI database under the project accession number SRP018875.

*De novo* assembly was carried out by SOAPdenovo, a genome assembly program developed specifically for next-generation short-read sequences [27]. The clean reads from the control-callus, salt-stressed callus and desert-grown tree sub-transcriptomes were assembled into 226 299, 200 532 and 1 497 985 contigs, respectively. The average contig size exceeded 101 bp in all three libraries (Table 1). The high heterozygosity of this wind-pollinated species may have resulted in the high number of contigs and the small length of contigs for desert-grown trees sample (Table 1). After using paired-end information to join the contigs into scaffolds and local assembly, we generated 119 471 scaffolds for control-callus samples, 106 812 scaffolds for salt-stressed callus samples and 161 908 scaffolds for desert-grown trees, with average lengths of 381, 392 and 226 nt, respectively. These scaffolds were assembled into 122 883 unigenes by TGICL clustering tools, more than those (94 196 unigenes) from *P. euphratica* [24]. Out of the 122 883 unigenes, 49 349 unigenes were ≥500 bp and 17 402 were ≥1,000 bp, with an average unigenes length of 526 bp and an N50 size of 681 bp (Table 1). The assembly of transcriptome sequencing reads produced more scaffolds than the expressed genes, suggesting the redundant assembled sequences (i.e., more than one sequence per gene). According to 73 013 transcripts in *P. trichocarpa* (based on *Populus trichocarpa* v3.0, DOE-JGI, http:://www.phytozome.net/poplar), the number of all-unigenes is clearly >1.5× more than the number of genes in a poplar species. The size distribution for these unigenes is shown in Figure S1, with the majority being shorter sequences. Numerous short sequences may result from the fragmented genes.

**Table 1:** Overview of the *de novo* assembly of the transcriptome of *P. pruinosa*.

Sequences	control-callus	salt-stressed callus	desert-grown trees
Contig			
Number of contigs	226 299	200 532	1 497 985
Length of all contigs (nt)	47 707 523	43 936 971	151 124 036
Average contig size	211	219	101
Range of contig length	75-3 357	75-3 970	75-2 331
N50 (bp) 1	259	274	90
Scaffolds			
Number of scaffolds	119 471	106 812	161 908
Length of all scaffolds (nt)	45 468 086	41 913 454	36 670 030
Average scaffold sizes	381	392	226
Range of scaffold lengths	100-4 165	100-5 569	100-2 331
N50 (bp)	567	598	249
All Unigenes sequences			
Number of Unigenes	122 883		
Length of all Unigenes (nt)	64 580 690		
Average Unigene size	526		
Range of Unigene length	200-5 569		
N50 (bp)	681		

1 N50 size is a weighted median statistic indicating that 50% of the entire assembly resides in contigs/scaffolds of a length at least X.

To evaluate the quality of the dataset, we analyzed the gap ratio of the assembly and the ratio of the gap's length to the length of all-unigenes (Figures S2 and S3). The majority of the unigenes showed gap lengths that were less than 5% of the total length, which accounted for 70.1% of the total unigenes (86 870 unigenes). In addition, sequencing bias was also analyzed by examining random distribution of reads in all-unigenes (Figure S4). All-unigenes were evenly covered by the reads from the control-callus and salt-stressed callus sub-transcriptomes with relatively fewer reads in the 3′ ends of them (Figures S4A and S4B). This observation is consistent with previous reports [40], [41], suggesting that the quality of our dataset was comparable to similar studies of the other non-model species despite a peak in the distribution of reads at 0.3 relative gene length (Figure S4C). However, this did not affect subsequent analyses after clustering the unigenes using the TGI Clustering Tool.

We excluded possible known non-coding RNAs, microbial, fungal and virus sequences identified by comparing our unigene sequences against entries in databases listed in the Materials and methods section. Finally, we identified a total of 114 866 high-quality unique sequences from *P. pruinosa* when 8 017 contaminated or confounded sequences were excluded.

### Functional Annotation

The entire unigene sets were then annotated on the basis of similarities to known or putative sequences in public databases. Among the 114 866 high-quality unique sequences, 67 400 (58.7%) had at least one significant match to an existing gene model in BLASTX searches (Table 2).

**Table 2:** Functional annotation of high-quality unique sequences by sequence similarity (e-value<1e-5).

	Database	*P. euphratica*		*P. pruinosa*	
		Number	Percent (%)	Number	Percent (%)
Annotated	Nr	58 314	67.2	65 219	56.8
	Swissprot	36 330	41.9	39 524	34.4
	COG	16 096	18.5	17 905	15.6
	KEGG	24 504	28.2	26 817	23.3
	GO	92 96	10.7	11 587	10.1
	Total	58 499	67.4	67 400	58.7
Unannotated		28 278	32.6	47 466	41.3
Total		86 777	100	114 866	100

Transcriptome annotation of *P. euphratica* was reported by Qiu et al [24]. GO was searched by Blast2GO.

**Figure 1:**
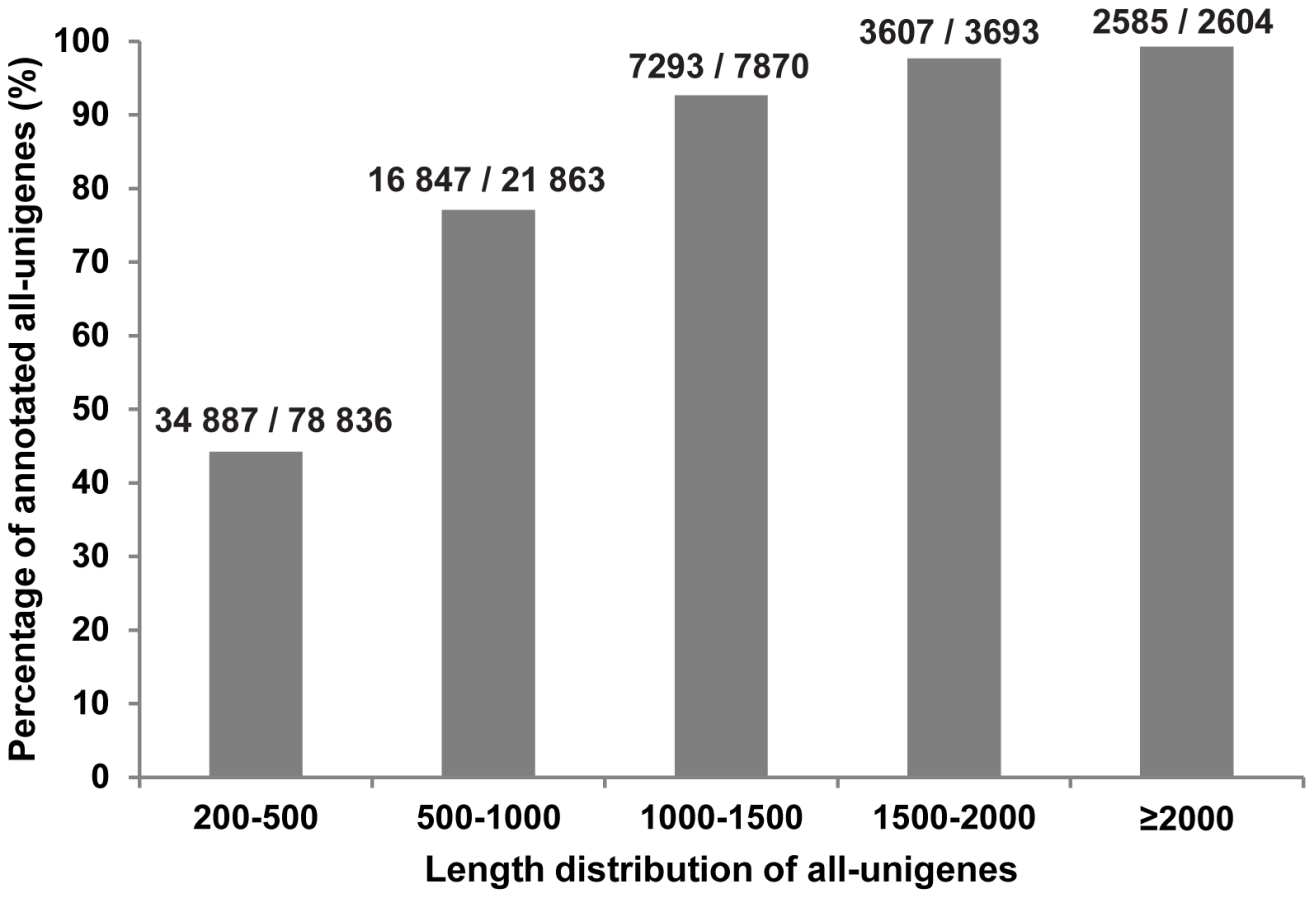
Length distribution of the all-unigenes with Nr annotations. More than 80% of the all-unigenes over 500 bp had BLAST hits in the Nr database.

**Figure 2:**
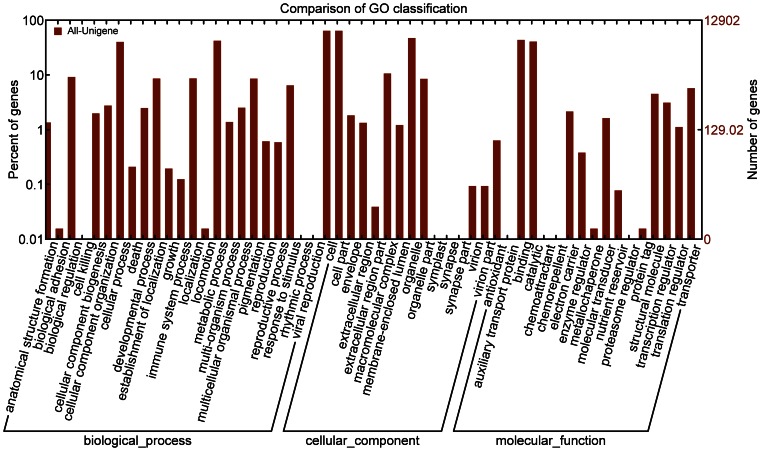
GO categories of the *P.* *pruinosa* all-unigenes. WEGO was used to produce the graph. The results are summarized in ‘biological process’, ‘cellular component’, and ‘molecular function’. The percentage (left y-axis) and total number (right y-axis) of all-unigenes in each category (the third GO level) are shown. In total, 11 587 all-unigenes have been assigned. Y axis is in log(10) scale.

First, the all-unigenes of *P. pruinosa* were assigned putative gene descriptions based on the BLAST search against the NCBI non-redundant (Nr) protein database. Out of the 114 866 all-unigenes, 65 219 (56.8%) showed significant similarity with proteins in the Nr database (Table 2). The proportion of all-unigenes with BLAST hits increased markedly for those with larger sizes (Figure 1). It seemed that longer all-unigenes were more likely to have Nr annotations. The E-value distribution of the top hits in the Nr database also revealed that a larger proportion of all-unigenes longer than 500 bp had strong homology (Figure S5). Following their Nr annotations, we mapped all-unigenes into the records of the GO database and retrieved GO annotations for 11 587 ones (10.1%) (Table 2). This observation of low proportion of all-unigenes assigned to a GO term was comparable to the previous reports [24], [41]. These all-unigenes were assigned into GO terms with three main functional categories, including 6 194 all-unigenes in ‘Biological process’, 7 409 in ‘Cellular component’, and 7 818 in ‘Molecular function’ (Figure 2 and Table S2) while 3 381 all-unigenes had an assignment in all three categories. The remaining all-unigenes failed to obtain a GO term, largely due to their uninformative (e.g. ‘unknown’, ‘putative’, or ‘hypothetical’ protein) descriptions. Within the ‘Biological process’, there were 21 GO categories and the two most abundantly represented lineages were ‘metabolic process’ and ‘cellular process’. There were also a large number of all-unigenes being involved in ‘biological regulation’, ‘localization’, ‘pigmentation’, and ‘response to stimulus’. In the ‘Molecular function’ division, there were 13 GO categories and the top two categories were ‘binding’ and ‘catalytic activity’. The former was mainly represented by genes for ‘nucleotide binding’ and ‘protein binding’, while the latter was mainly represented by genes with ‘transferase activity’, ‘hydrolase activity’, and‘kinase activity’. Finally, in the ‘Cellular component’ division, there were 11 categories and the top two categories being ‘cell’ and ‘organelle’. The matched proportions of GO categories in *P. pruinosa* ESTs are similar to those for *P. euphratica* [24], suggesting that our library and Illumina sequencing have adequately sampled the species' total transcriptome.

### Identification of Putative Orthologs Between *P. Pruinosa* and *P. Euphratica*

We recovered 68 526 CDSs for *P. pruinosa*, more than those recovered (59 721) from *P. euphratica* [24]. The average CDSs length for the *P. pruinosa* and *P. euphratica* were 437 and 532 respectively (Table S3). We identified 23 167 pairs of orthologs between *P. pruinosa* and *P. euphratica*, through a reciprocal best hit blast search. The orthologous distributions (Figure S6) suggested that most non-orthologous pairs were excluded. After a series of strict filtration, we then obtained 2859 pairs of transcripts that are highly orthologous between species. The median length of these transcripts is 1079 bp, ranging from 573 to 3555 bp (Table S3).

### *Ka/Ks* Between Pairs of Orthologs

**Figure 3:**
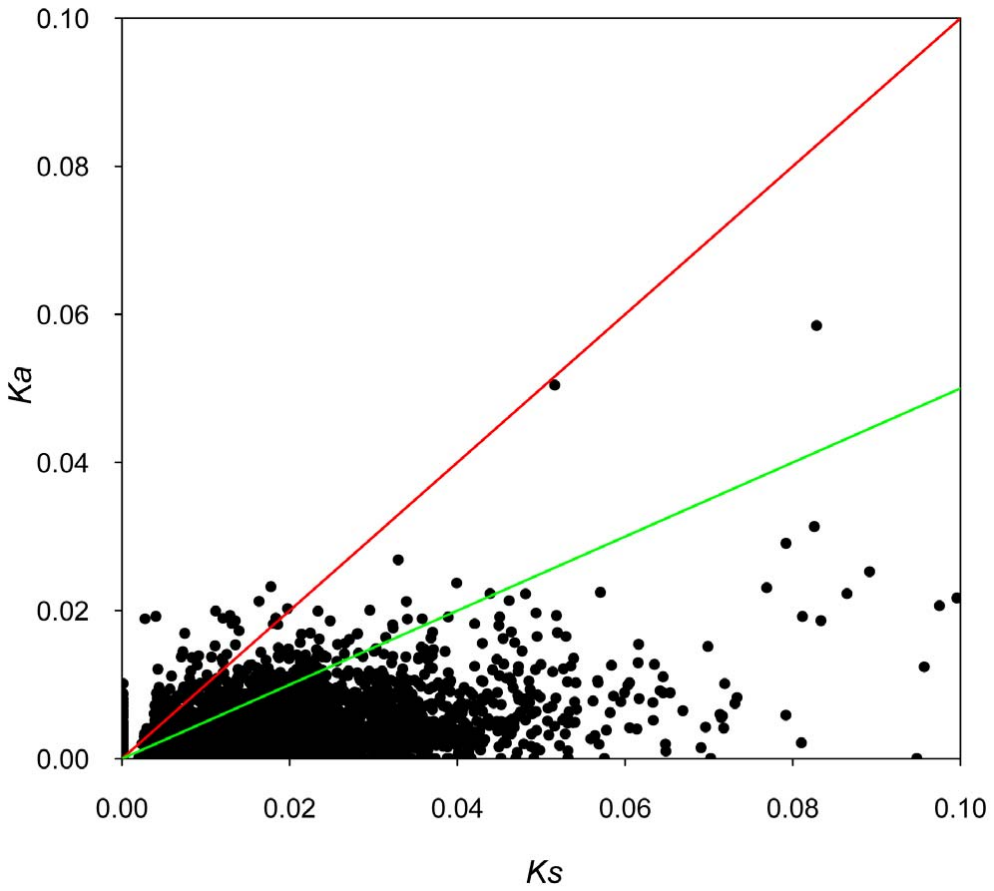
Distribution of *Ka* and *Ks* for 2 859 pairs of the putative orthologs. The orthologs with *Ka/Ks*>1 fall above the red line while those with *Ka/Ks* = 0.5−1 fall between the green and red lines.

Out of the 2859 ortholog pairs, divergence was sufficiently high for 2339 orthologs (81.8%) to allow both *Ka* and *Ks* rates to be calculated (Table S4). Some of them showed high *Ka/Ks* values. Of these, 84 pairs of orthologs (2.9%) have a *Ka/Ks* >1, indicating positive selection, and 355 pairs (12.4%) have a *Ka/Ks* between 0.5 and 1, indicating weak purifying selection (Figure 3). The average synonymous substitutions and nonsynonymous substitutions for these 84 ortholog pairs were 2 and 8, respectively. For the remaining pairs of orthologs, we could calculate either only *Ka* (332 pairs of orthologs, 11.6%), or *Ks* (99 pairs of orthologs, 3.5%), or incalculable estimations (89 pairs of orthologs or 3.1%) (Table S4). The functions of the homologous genes of those pairs of orthologs with *Ka/Ks*>1 were mainly involved in ‘responses to stress’, ‘ubiquitin-dependent protein catabolic processes’, and ‘biological regulation’ (Table S5 and Table 3).

**Table 3:** GO categories over-represented among the homologous genes of the orthologs with positive selection (P-values from the Fisher's exact test).

GO categories	Description	Taxonomy	P-Value
GO:0019941	modification-dependent protein catabolic process	P	0.02846
GO:0006511	ubiquitin-dependent protein catabolic process	P	0.02846
GO:0043632	modification-dependent macromolecule catabolic process	P	0.02846
GO:0006914	autophagy	P	0.02933
GO:0006508	proteolysis	P	0.03312
GO:0042991	transcription factor import into nucleus	P	0.04625
GO:0048364	root development	P	0.04786
GO:0043901	negative regulation of multi-organism process	P	0.04944
GO:1900366	negative regulation of defense response to insect	P	0.04944
GO:0002832	negative regulation of response to biotic stimulus	P	0.04944
GO:0010102	lateral root morphogenesis	P	0.04944
GO:2000068	regulation of defense response to insect	P	0.04944
GO:0009231	riboflavin biosynthetic process	P	0.04944
GO:0010101	post-embryonic root morphogenesis	P	0.04944
GO:0042727	flavin-containing compound biosynthetic process	P	0.04944
GO:0047213	anthocyanidin 3-O-glucosyltransferase activity	F	0.01567
GO:0003677	DNA binding	F	0.03945
GO:0044183	protein binding involved in protein folding	F	0.04944
GO:0042409	caffeoyl-CoA O-methyltransferase activity	F	0.04944
GO:0000062	fatty-acyl-CoA binding	F	0.04944
GO:0004156	dihydropteroate synthase activity	F	0.04944
GO:0003848	2-amino-4-hydroxy-6-hydroxymethyldihydropteridine diphosphokinase activity	F	0.04944
GO:0060090	binding, bridging	F	0.04944
GO:0004024	alcohol dehydrogenase activity, zinc-dependent	F	0.04944
GO:0008835	diaminohydroxyphosphoribosylaminopyrimidine deaminase activity	F	0.04944
GO:0032791	lead ion binding	F	0.04944
GO:0019786	APG8-specific protease activity	F	0.04944
GO:0016807	cysteine-type carboxypeptidase activity	F	0.04944
GO:0070004	cysteine-type exopeptidase activity	F	0.04944
GO:0005775	vacuolar lumen	C	0.04944
GO:0009514	glyoxysome	C	0.04944
GO:0000502	proteasome complex	C	0.04944
GO:0005776	autophagic vacuole	C	0.04944

Gene ontology: F, molecular function, P, biological process, C, cellular components.

For those pairs of ESTs with the sign of adaptive evolution, we randomly selected 10 pairs and designed the starting and ending primers across two species. The adaptive mutations were confirmed by amplifying and sequencing five individuals of each species (Figure S7). In addition, for each locus, all sequences from each species clustered respectively, similarly suggesting that all pairs of ESTs for analyses are orthologous rather than paralogous. For each pair of orthologs, most mutations indicated by EST comparisons were confirmed as species-specific; however, a few of them were found to be ‘introgressed’ into the other from one species (for example, Unigene2702_All, site 190, Figure S7). In addition, we found that all of five individuals in one species possessed additive polymorphisms at some sites while those of the other species were fixed for one of two additive polymorphisms (for example, Unigene37561_All, site 607, additive polymorphisms W = A/T for *P. euphratica* and only ‘A’ for *P. pruinosa*, FigureS7).

### Expression Changes of Rapidly Evolved Orthologs Under the Salt Stress

**Figure 4:**
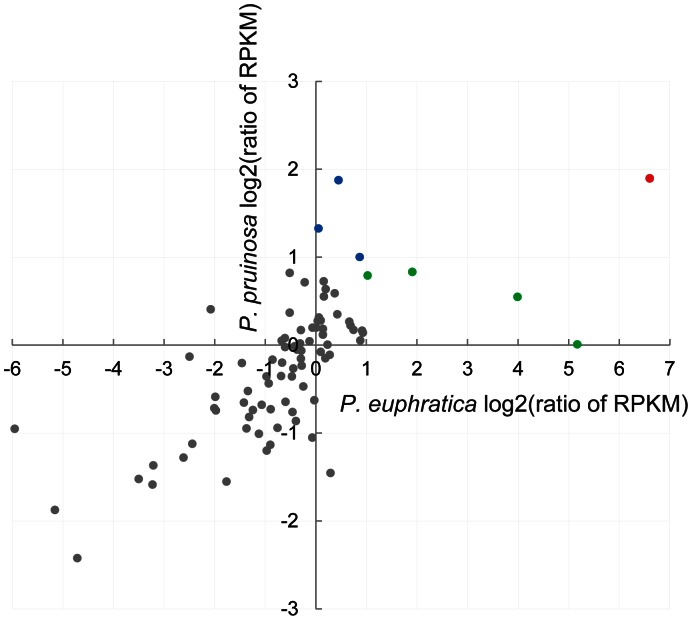
Gene expressions of 84 pairs of orthologs in *P.* *euphratica* and *P. pruinosa* under salt stress. Red dot – higher expression levels in both *P. euphratica* and *P. pruinosa*; green dots – higher expression levels only in *P. euphratica*; and blue dots – higher expression levels only in *P. pruinosa*.

For the positively selected genes, gene expression levels were measured and RPKM values were generated by mapping the reads from the control and salt-stressed callus subtranscriptomes to it for *P. euphratica* and *P. pruinosa* (Table S6). Based on the log 2 ratio value, we found that only one pair of the orthologous ESTs (Unigene56801_All and Unigene25389_All in *P. euphratica* and *P. pruinosa*, homologous to *Arabidopsis* Proteinase inhibitor I4, serpin) has a higher expression level in salt-stressed callus when compared with control callus in both *P. euphratica* and *P. pruinosa*. Four pairs of orthologous ESTs (Unigene7552_All, Unigene65280_All, Unigene5742_All and Unigene34836_All in *P. euphratica*, homologous to *Arabidopsis* UDP-glucose:glucosyltransferase, glycosyltransferase, wax synthase isoform 3, and Ripening-related protein grip22) have higher expression levels in salt-stress callus when compared with control callus in *P. euphratica* but not in *P. pruinosa*. Three pairs of orthologous ESTs (Unigene45594_All, Unigene5696_All and Unigene18546_All in *P. pruinosa*, homologous to Arabidopsis *MYB* family transcription factor, WAK-like kinase, and NAC domain protein, IPR003441 (NAC036)) have higher expression levels in salt-stress callus when compared with control callus in *P. pruinosa* in but not in *P. euphratica* (Figure 4).

## Discussion

In this study, over 85 million sequencing reads from various samples were generated and assembled into a total of 114 866 high-quality unique sequences for the *P. pruinosa* transcriptome. The low level of contamination with organelle DNA and the in depth coverage of genes involved in various biological processes indicate that ‘next generation’ sequencing is an excellent tool for the gene discovery, EST sequencing, and transcriptome analyses in the non-model tree species [42].

Based on comparative genomic analysis of expressed sequence tags (ESTs) from two sister poplar species, *P. pruinosa* and *P. euphratica*, which diverged in the recent past, our study confirmed the initial expectation that genes (represented by these orthologous ESTs) with signs of positive selection are closely related to selective adaptation of the two species into different desert habitats. A total of 84 (2.9%) pairs of orthologs have *Ka/Ks*>1, exhibiting rapid evolution with signs of strong selection. It should be noted that our strict criteria may have filtered some candidates with rapid evolutions. In addition, our results based on sequencing more individuals of each species for a few selected orthologs suggested that most mutations are species-specific possibly due to adaptive differentiations although interspecific gene flow between *P. euphratica* and *P. pruinosa* [20] may have resulted in the introgressions and/or additive polymorphisms at a few sites of these genes (Figure S7).

It is interesting that most of these recovered ESTs showing rapid evolution are not related to reproductive isolation. Because our sampling could not encompass adaptive evolution of all putative genes from both species, we could not rule out the possibility that other genes related to reproductive isolation were over-looked in this study. It is particularly important that genes homologous to the detected pairs of ESTs with rapid evolution are significantly enriched in ‘responses to stress’, ‘ubiquitin-dependent protein catabolic processes’, and ‘biological regulation’ (Table S5 and Table 3). For example, one of the genes classified under “responses to stress”, *RD22* (homologues of Unigene36416_All for *P. pruinosa* and Unigene15370_All for *P. euphratica*), plays an important role in regulating abiotic tolerance against oxidative and drought stresses [43]. Another rapidly evolving gene, *RAD23* (homologues of Unigene106_All for *P. pruinosa* and Unigene31797_All for *P. euphratica*), takes part in the Ubiquitin-dependent protein catabolic process in most aspects of a plant's life cycle [44]-[46]. In order to adapt differentiated salty habitats [47], [48], therefore, the genes at the same category or network seem to have evolved rapidly.

However, it should be noted that not all detected pairs of ESTs with positive selection showed the same expression changes in response to salt stress (Table S6). We found only eight pairs of ESTs (around 10%) that changed expression patterns in either or both of the two species under salt stress. One pair of rapidly evolving ESTs experienced common up-regulation under salt stress in both species. However, four orthologous ESTs had higher expression levels in salt-stress callus of *P. euphratica* but not in that of *P. pruinosa*, while the reverse was true of the other three orthologous ESTs. These findings suggest that there is a basic pool of salt-responsive genes common to both species and that only some of them evolved rapidly during speciation. However, it is also clear that these two species underwent independent selection for salt adaptation because the remaining seven pairs of orthologous ESTs detected changed their expression only in one species. Although both species grow in salty deserts, *P. euphratica* occurs in the dryer deserts with the deep underground water while *P. pruinosa* in deserts with underground water closer to the surface. In response to these differentiated salty habitats, the two species may have adopted different genes belonging to the same category or pathway to cope with salt stress, which may have finally triggered their respective rapid evolutions. In addition, multiple pathways or genetic networks are involved in such a stress response [49]. Interactions between salt and other stresses could have also exacerbated rapid evolution of some genes at the salt-related pathways in the two poplar species. Thus, our findings suggest that the genes with differentiated expressions or functions at the same category or network may have evolved rapidly during adaptive divergence of two species to differentiated salty desert habitats.

Finally, it should be noted that our results show that comparing orthologous ESTs between closely related tree species by NGS transcriptome sequencing provides a fast and cost-effective approach to detect rapidly evolved genes and understand adaptive divergences of trees at genomic level to arid habitats. Further studies are needed to validate functional changes between these orthologous genes which we have recovered, especially in regard to interactions and correlations across different metabololic pathways related to salt stress.

## Supporting Information

Figure S1 **Length distribution of assembled scaffolds and Unigenes.**
(DOCX)

Figure S2 **Distribution of Gap (N) size for assembled scaffolds and Unigenes.**
(DOCX)

Figure S3 **Ratio distribution of the gap's length to the length of all-unigenes.** The x-axis indicates the ratio of the gap's length to the length of all-unigenes. The y-axis indicates the number of unigenes containing gaps.
(DOCX)

Figure S4 **Distribution of the reads from three sub-transcriptomes in the all-unigenes.** (A) control-callus, (B) salt-stressed callus, and (C) desert-grown trees. The x-axis indicates the relative position of sequencing reads in the all-unigenes. The orientation of the all-unigene is from 5′ end to 3′ end.
(DOCX)

Figure S5 **The E-value distribution of the top hits in the Nr database for two sets of all-unigenes.** (A) The total all-unigenes; (B) The all-unigenes longer than 500 bp.
(DOCX)

Figure S6 **The filtered orthologous distribution by the strict filtering criteria.** The total orthologous was 23 167. After a series of strict filtration, we obtained 2859 pairs of transcripts that are highly orthologous between species ultimately. The i, ii, iii, iv and v were represented filtering methods: i: corresponding best hits for the alignment of two ESTs that had a length overlap <150 bp; ii: ortholog pairs with a percent coverage <90%; iii: aligned sequences with unexpected stop-codons and ambiguous alignments; iv: orthologs with a synonymous (Ks) substitution value greater than 0.1; v: alignments were checked manually for errors.
(DOCX)

Figure S7 **10 individuals of the two species were amplified and sequenced using 10 pairs of primers.**
(PDF)

Table S1 **Output Statistics of the sequencing.**
(DOCX)

Table S2 **GO categories of the** ***P. pruinosa*** **all-unigenes based on Blast2GO.**
(DOCX)

Table S3 **Orthologous pairs and alignment summary.**
(DOCX)

Table S4 **KaKs Calculator was used to calculate** ***Ka*** **and** ***Ks*** **for the 2859 ortholog pairs.**
(XLSX)

Table S5 **Annotation information for the 84 ortholog pairs with** ***Ka/Ks*** **>1.**
(DOCX)

Table S6 **Gene expression levels for the positively selected genes.**
(XLSX)
