# Supplementary material for: Rapidly Evolving Genes and Stress Adaptation of Two Desert Poplars, Populus euphratica and P. pruinosa
Source: PLoS One. 2013 Jun 11;8(6):e66370. doi: 10.1371/journal.pone.0066370 (PMC3679102; doi:10.1371/journal.pone.0066370)
Supplement: Figure S2 — Distribution of Gap (N) size for assembled scaffolds and Unigenes. (DOCX) [file pone.0066370.s002.docx]

**Figure S2 Distribution of Gap (N) size for assembled scaffolds and Unigenes**
