# Supplementary material for: Rapidly Evolving Genes and Stress Adaptation of Two Desert Poplars, Populus euphratica and P. pruinosa
Source: PLoS One. 2013 Jun 11;8(6):e66370. doi: 10.1371/journal.pone.0066370 (PMC3679102; doi:10.1371/journal.pone.0066370)
Supplement: Figure S3 — Ratio distribution of the gap's length to the length of all-unigenes. The x-axis indicates the ratio of the gap's length to the length of all-unigenes. The y-axis indicates the number of unigenes containing gaps. (DOCX) [file pone.0066370.s003.docx]

**Figure S3 Ratio distribution of the gap's length to the length of all-unigenes.** The x-axis indicates the ratio of the gap's length to the length of all-unigenes. The y-axis indicates the number of unigenes containing gaps.
