# Supplementary material for: Rapidly Evolving Genes and Stress Adaptation of Two Desert Poplars, Populus euphratica and P. pruinosa
Source: PLoS One. 2013 Jun 11;8(6):e66370. doi: 10.1371/journal.pone.0066370 (PMC3679102; doi:10.1371/journal.pone.0066370)
Supplement: Figure S4 — Distribution of the reads from three sub-transcriptomes in the all-unigenes. (A) control-callus, (B) salt-stressed callus, and (C) desert-grown trees. The x-axis indicates the relative position of sequencing reads in the all-unigenes. The orientation of the all-unigene is from 5′ end to 3′ end. (DOCX) [file pone.0066370.s004.docx]

**Figure S4** **Distribution of the reads from three sub-transcriptomes in the all-unigenes.** (A) control-callus, (B) salt-stressed callus, and (C) desert-grown trees. The x-axis indicates the relative position of sequencing reads in the all-unigenes. The orientation of the all-unigene is from 5’ end to 3’ end.
