# Supplementary material for: Rapidly Evolving Genes and Stress Adaptation of Two Desert Poplars, Populus euphratica and P. pruinosa
Source: PLoS One. 2013 Jun 11;8(6):e66370. doi: 10.1371/journal.pone.0066370 (PMC3679102; doi:10.1371/journal.pone.0066370)
Supplement: Figure S5 — The E-value distribution of the top hits in the Nr database for two sets of all-unigenes. (A) The total all-unigenes; (B) The all-unigenes longer than 500 bp. (DOCX) [file pone.0066370.s005.docx]

**Figure S5 The E-value distribution of the top hits in the Nr database for two sets of all-unigenes.** (A) The whole of all-unigenes; (B) The all-unigenes longer than 500 bp.
