# Supplementary material for: Rapidly Evolving Genes and Stress Adaptation of Two Desert Poplars, Populus euphratica and P. pruinosa
Source: PLoS One. 2013 Jun 11;8(6):e66370. doi: 10.1371/journal.pone.0066370 (PMC3679102; doi:10.1371/journal.pone.0066370)
Supplement: Figure S6 — The filtered orthologous distribution by the strict filtering criteria. The total orthologous was 23 167. After a series of strict filtration, we obtained 2859 pairs of transcripts that are highly orthologous between species ultimately. The i, ii, iii, iv and v were represented filtering methods: i: corresponding best hits for the alignment of two ESTs that had a length overlap <150 bp; ii: ortholog pairs with a percent coverage <90%; iii: aligned sequences with unexpected stop-codons and ambiguous alignments; iv: orthologs with a synonymous (Ks) substitution value greater than 0.1; v: alignments were checked manually for errors. (DOCX) [file pone.0066370.s006.docx]

**Figure S6 The filtered orthologous distribution by the strict filtering criteria.**

The total orthologous was 23 167. After a series of strict filtration, we obtained 2859 pairs of transcripts that are highly orthologous between species ultimately. The i, ii, iii, iv and v were represented filtering methods: i: corresponding best hits for the alignment of two ESTs that had a length overlap < 150 bp; ii: ortholog pairs with a percent coverage < 90%; iii: aligned sequences with unexpected stop-codons and ambiguous alignments; iv: orthologs with a synonymous (Ks) substitution value greater than 0.1; v: alignments were checked manually for errors.
