# Supplementary material for: Rapidly Evolving Genes and Stress Adaptation of Two Desert Poplars, Populus euphratica and P. pruinosa
Source: PLoS One. 2013 Jun 11;8(6):e66370. doi: 10.1371/journal.pone.0066370 (PMC3679102; doi:10.1371/journal.pone.0066370)
Supplement: Figure S7 — 10 individuals of the two species were amplified and sequenced using 10 pairs of primers. (PDF) [file pone.0066370.s007.pdf]

Unigene20083_All (DNA-binding protein DF1)

```
              ....|....| ....|....| ....|....| ....|....| ....|....| ....|....| ....|....|
                      10         20         30         40         50         60         70
Peu_1         AACCCTACAA ATCCTCCTCA TACTAATATT TCACCCTCTT TTCCAAACTT CTCCTCAGAT CTCATCTCCA
Peu_2         AACCCTACAA ATCCTCCTCA TACTAATATT TCACCCTCTT TTCCAAACTT CTCCTCAGAT CTCATCTCCA
Peu_3         AACCCTACAA ATCCTCCTCA TACTAATATT TCACCCTCTT TTCCAAACTT CTCCTCAGAT CTCATCTCCA
Peu_4         AACCCTACAA ATCCTCCTCA TACTAATATT TCACCCTCTT TTCCAAACTT CTCCTCAGAT CTCATCTCCA
Peu_5         AACCCTACAA ATCCTCCTCA TACTAATATT TCACCCTCTT TTCCAAACTT CTCCTCAGAT CTCATCTCCA
Ppr_1         AACCCTACAA ATCCTCCTCA TACTAATATT CCACCCTCTT TTCCAAACTT CTCCTCAGAT CTCATCTCCA
Ppr_2         AACCCTACAA ATCCTCCTCA TACTAATATT CCACCCTCTT TTCCAAACTT CTCCTCAGAT CTCATCTCCA
Ppr_3         AACCCTACAA ATCCTCCTCA TACTAATATT CCACCCTCTT TTCCAAACTT CTCCTCAGAT CTCATCTCCA
Ppr_4         AACCCTACAA ATCCTCCTCA TACTAATATT CCACCCTCTT TTCCAAACTT CTCCTCAGAT CTCATCTCCA
Ppr_5         AACCCTACAA ATCCTCCTCA TACTAATATT CCACCCTCTT TTCCAAACTT CTCCTCAGAT CTCATCTCCA

              ....|....| ....|....| ....|....| ....|....| ....|....| ....|....| ....|....|
                      80         90        100        110        120        130        140
Peu_1         ATTCCACTTC TTCATCGACA TCCTCGGACG TGGAACTGCA AGAGCGTTGC AAGAGGAAAA GGAAATGGAA
Peu_2         ATTCCACTTC TTCATCGACA TCCTCGGACG TGGAACTGCA AGAGCGTTGC AAGAGGAAAA GGAAATGGAA
Peu_3         ATTCCACTTC TTCATCGACA TCCTCGGACG TGGAACTGCA AGAGCGTTGC AAGAGGAAAA GGAAATGGAA
Peu_4         ATTCCACTTC TTCATCGACA TCCTCGGACG TGGAACTGCA AGAGCGTTGC AAGAGGAAAA GGAAATGGAA
Peu_5         ATTCCACTTC TTCATCGACA TCCTCGGACG TGGAACTGCA AGAGCGTTGC AAGAGGAAAA GGAAATGGAA
Ppr_1         ATTCCACTTC TTCATCGACA TCCTCGGACG TGGAACTGCA AGAGCGTTGC AAGAGGAAAA GGAAATGGAA
Ppr_2         ATTCCACTTC TTCATCGACA TCCTCGGACG TGGAACTGCA AGAGCGTTGC AAGAGGAAAA GGAAATGGAA
Ppr_3         ATTCCACTTC TTCATCGACA TCCTCGGACG TGGAACTGCA AGAGCGTTGC AAGAGGAAAA GGAAATGGAA
Ppr_4         ATTCCACTTC TTCATCGACA TCCTCGGACG TGGAACTGCA AGAGCGTTGC AAGAGGAAAA GGAAATGGAA
Ppr_5         ATTCCACTTC TTCATCGACA TCCTCGGACG TGGAACTGCA AGAGCGTTGC AAGAGGAAAA GGAAATGGAA

              ....|....| ....|....| ....|....| ....|....| ....|....| ....|....| ....|....|
                     150        160        170        180        190        200        210
Peu_1         GGACTTCTTT GAAAGGCTCA TGAAGGAGGT GATCCAGAAG CAAGAGGAGA TGCAGAAGAA GTTCTTGGAA
Peu_2         GGACTTCTTT GAAAGGCTCA TGAAGGAGGT GATCCAGAAG CAAGAGGAGA TGCAGAAGAA GTTCTTGGAA
Peu_3         GGACTTCTTT GAAAGGCTCA TGAAGGAGGT GATCCAGAAG CAAGAGGAGA TGCAGAAGAA GTTCTTGGAA
Peu_4         GGACTTCTTT GAAAGGCTCA TGAAGGAGGT GATCCAGAAG CAAGAGGAGA TGCAGAAGAA GTTCTTGGAA
Peu_5         GGACTTCTTT GAAAGGCTCA TGAAGGAGGT GATCCAGAAG CAAGAGGAGA TGCAGAAGAA GTTCTTGGAA
Ppr_1         GGACTTCTTT GAAAGGCTCA TGAAGGAGGT GATCCAGAAG CAAGAGGAGA TGCAGAAGAA GTTCTTGGAA
Ppr_2         GGACTTCTTT GAAAGGCTCA TGAAGGAGGT GATCCAGAAG CAAGAGGAGA TGCAGAAGAA GTTCTTGGAA
Ppr_3         GGACTTCTTT GAAAGGCTCA TGAAGGAGGT GATCCAGAAG CAAGAGGAGA TGCAGAAGAA GTTCTTGGAA
Ppr_4         GGACTTCTTT GAAAGGCTCA TGAAGGAGGT GATCCAGAAG CAAGAGGAGA TGCAGAAGAA GTTCTTGGAA
Ppr_5         GGACTTCTTT GAAAGGCTCA TGAAGGAGGT GATCCAGAAG CAAGAGGAGA TGCAGAAGAA GTTCTTGGAA

              ....|....| ....|....| ....|....| ....|....| ....|....| ....|....| ....|....|
                     220        230        240        250        260        270        280
Peu_1         GCAATTGAGA GACTCGAACA TGAAAGAATG GTTAGAGAAG AATCTTGGAG AATGCAGGAA ATGACAAGAA
Peu_2         GCAATTGAGA GACTCGAACA TGAAAGAATG GTTAGAGAAG AATCTTGGAG AATGCAGGAA ATGACAAGAA
Peu_3         GCAATTGAGA GACTCGAACA TGAAAGAATG GTTAGAGAAG AATCTTGGAG AATGCAGGAA ATGACAAGAA
Peu_4         GCAATTGAGA GACTCGAACA TGAAAGAATG GTTAGAGAAG AATCTTGGAG AATGCAGGAA ATGACAAGAA
Peu_5         GCAATTGAGA GACTCGAACA TGAAAGAATG GTTAGAGAAG AATCTTGGAG AATGCAGGAA ATGACAAGAA
Ppr_1         GCAATTGAGA GACGCGAACA TGAAAGAATG GTTAGAGAAG AATCTTGGAG AACGCAGGAA ATGACAAGAA
Ppr_2         GCAATTGAGA GACGCGAACA TGAAAGAATG GTTAGAGAAG AATCTTGGAG AACGCAGGAA ATGACAAGAA
Ppr_3         GCAATTGAGA GACGCGAACA TGAAAGAATG GTTAGAGAAG AATCTTGGAG AACGCAGGAA ATGACAAGAA
Ppr_4         GCAATTGAGA GACGCGAACA TGAAAGAATG GTTAGAGAAG AATCTTGGAG AACGCAGGAA ATGACAAGAA
Ppr_5         GCAATTGAGA GACGCGAACA TGAAAGAATG GTTAGAGAAG AATCTTGGAG AACGCAGGAA ATGACAAGAA

              ....|....| ....|....| ....|....| ....|....| ....|....| ....|....| ....|....|
                     290        300        310        320        330        340        350
Peu_1         TCAATAGAGA GAGCGAGATC TTAGCCCAAG AAAGATCCGT TGCGGCAAGT AAAAATGCTG AGGTAATGGC
Peu_2         TCAATAGAGA GAGCGAGATC TTAGCCCAAG AAAGATCCGT TGCGGCAAGT AAAAATGCTG AGGTAATGGC
Peu_3         TCAATAGAGA GAGCGAGATC TTAGCCCAAG AAAGATCCGT TGCGGCAAGT AAAAATGCTG AGGTAATGGC
Peu_4         TCAATAGAGA GAGCGAGATC TTAGCCCAAG AAAGATCCGT TGCGGCAAGT AAAAATGCTG AGGTAATGGC
Peu_5         TCAATAGAGA GAGCGAGATC TTAGCCCAAG AAAGATCCGT TGCGGCAAGT AAAAATGCTG AGGTAATGGC
Ppr_1         TCAATAGAGA GCGCGAGATC TTAGCCCAAG AAAGATCCGC TGCGACAAGT ATAGATGCTG CGGTAATGGC
Ppr_2         TCAATAGAGA GCGCGAGATC TTAGCCCAAG AAAGATCCGC TGCGACAAGT ATAGATGCTG CGGTAATGGC
Ppr_3         TCAATAGAGA GCGCGAGATC TTAGCCCAAG AAAGATCCGC TGCGACAAGT ATAGATGCTG CGGTAATGGC
Ppr_4         TCAATAGAGA GCGCGAGATC TTAGCCCAAG AAAGATCCGC TGCGACAAGT ATAGATGCTG CGGTAATGGC
Ppr_5         TCAATAGAGA GCGCGAGATC TTAGCCCAAG AAAGATCCGC TGCGACAAGT ATAGATGCTG CGGTAATGGC

              ....|....| ....|....| ....|....| ....|....| ....|....| ...
                     360        370        380        390        400
```

Peu_1 ATTTTTGCAA AAATTATCCG AAGAGCAACA TCCGGGTCAA ATACAAAATA ATC
Peu_2 ATTTTTGCAA AAATTATCCG AAGAGCAACA TCCGGGTCAA ATACAAAATA ATC
Peu_3 ATTTTTGCAA AAATTATCCG AAGAGCAACA TCCGGGTCAA ATACAAAATA ATC
Peu_4 ATTTTTGCAA AAATTATCCG AAGAGCAACA TCCGGGTCAA ATACAAAATA ATC
Peu_5 ATTTTTGCAA AAATTATCCG AAGAGCAACA TCCGGGTCAA ATACAAAATA ATC
Ppr_1 ATTTTTGCAA AAATTATCCG AAGAGCAACA TCCGGTTCAA ATACAAAATA ATC
Ppr_2 ATTTTTGCAA AAATTATCCG AAGAGCAACA TCCGGTTCAA ATACAAAATA ATC
Ppr_3 ATTTTTGCAA AAATTATCCG AAGAGCAACA TCCGGTTCAA ATACAAAATA ATC
Ppr_4 ATTTTTGCAA AAATTATCCG AAGAGCAACA TCCGGTTCAA ATACAAAATA ATC
Ppr_5 ATTTTTGCAA AAATTATCCG AAGAGCAACA TCCGGTTCAA ATACAAAATA ATC

Unigene26469_All (pentatricopeptide (PPR) repeat-containing protein)

```
                ....|....| ....|....| ....|....| ....|....| ....|....| ....|....| ....|....|
                          10         20         30         40         50         60         70
Peu_1           TTGTCC-ACC CACTACACCT TTGGGAACCT GCTGAATGCC TCTGCAAATC TTGCTGATCT GAAGCTTGGC
Peu_2           TTGTCC-ACC CACTACACCT TTGGGAACCT GCTGAATGCC TCTGCAAATC TTGCTGATCT GAAGCTTGGC
Peu_3           TTGTCC-ACC CACTACACCT TTGGGAACCT GCTGAATGCC TCTGCAAATC TTGCTGATCT GAAGCTTGGC
Peu_4           TTGTCC-ACC CACTACACCT TTGGGAACCT GCTGAATGCC TCTGCAAATC TTGCTGATCT GAAGCTTGGC
Peu_5           TTGTCC-ACC CACTACACCT TTGGGAACCT GCTGAATGCC TCTGCAAATC TTGCTGATCT GAAGCTTGGC
Ppr_1           TTGTCCGACC CACTACACCT TTGGGAACCT GCTGAATGCC TCTGCAAATC TTGCTGATCT GAAGCTTGGC
Ppr_2           TTGTCCGACC CACTACACCT TTGGGAACCT GCTGAATGCC TCTGCAAATC TTGCTGATCT GAAGCTTGGC
Ppr_3           TTGTCCGACC CACTACACCT TTGGGAACCT GCTGAATGCC TCTGCAAATC TTGCTGATCT GAAGCTTGGC
Ppr_4           TTGTCCGACC CACTACACCT TTGGGAACCT GCTGAATGCC TCTGCAAATC TTGCTGATCT GAAGCTTGGC
Ppr_5           TTGTCCGACC CACTACACCT TTGGGAACCT GCTGAATGCC TCTGCAAATC TTGCTGATCT GAAGCTTGGC

                ....|....| ....|....| ....|....| ....|....| ....|....| ....|....| ....|....|
                          80         90        100        110        120        130        140
Peu_1           AGGCAGGCAC ATAGTCATGT TGTTAAGCAT GGATTTAGGT TCCAGTCTGG TGAAGAGCCT GATATTTTTG
Peu_2           AGGCAGGCAC ATAGTCATGT TGTTAAGCAT GGATTTAGGT TCCAGTCTGG TGAAGAGCCT GATATTTTTG
Peu_3           AGGCAGGCAC ATAGTCATGT TGTTAAGCAT GGATTTAGGT TCCAGTCTGG TGAAGAGCCT GATATTTTTG
Peu_4           AGGCAGGCAC ATAGTCATGT TGTTAAGCAT GGATTTAGGT TCCAGTCTGG TGAAGAGCCT GATATTTTTG
Peu_5           AGGCAGGCAC ATAGTCATGT TGTTAAGCAT GGATTTAGGT TCCAGTCTGG TGAAGAGCCT GATATTTTTG
Ppr_1           AGGCAGGCAC ATAGTCATGT TGTTAAGCAT GGATTTAGGT TCCAGTCTGG TGAAGAGCCT GATATTTTTG
Ppr_2           AGGCAGGCAC ATAGTCATGT TGTTAAGCAT GGATTTAGGT TCCAGTCTGG TGAAGAGCCT GATATTTTTG
Ppr_3           AGGCAGGCAC ATAGTCATGT TGTTAAGCAT GGATTTAGGT TCCAGTCTGG TGAAGAGCCT GATATTTTTG
Ppr_4           AGGCAGGCAC ATAGTCATGT TGTTAAGCAT GGATTTAGGT TCCAGTCTGG TGAAGAGCCT GATATTTTTG
Ppr_5           AGGCAGGCAC ATAGTCATGT TGTTAAGCAT GGATTTAGGT TCCAGTCTGG TGAAGAGCCT GATATTTTTG

                ....|....| ....|....| ....|....| ....|....| ....|....| ....|....| ....|....|
                         150        160        170        180        190        200        210
Peu_1           TGGGAAATTC TCTCATAGAC ATGTATATGA AATGTGGATC TGTTGAAGAG GGACTTCGGG TTTTTGAAAA
Peu_2           TGGGAAATTC TCTCATAGAC ATGTATATGA AATGTGGATC TGTTGAAGAG GGACTTCGGG TTTTTGAAAA
Peu_3           TGGGAAATTC TCTCATAGAC ATGTATATGA AATGTGGATC TGTTGAAGAG GGACTTCGGG TTTTTGAAAA
Peu_4           TGGGAAATTC TCTCATAGAC ATGTATATGA AATGTGGATC TGTTGAAGAG GGACTTCGGG TTTTTGAAAA
Peu_5           TGGGAAATTC TCTCATAGAC ATGTATATGA AATGTGGATC TGTTGAAGAG GGACTTCGGG TTTTTGAAAA
Ppr_1           TGGGAAATTC TCTCATAGAC ATGTATATGA AATGTGGATC TGTTGAAGAG GGACTTCGGG TTTTTGAAAA
Ppr_2           TGGGAAATTC TCTCATAGAC ATGTATATGA AATGTGGATC TGTTGAAGAG GGACTTCGGG TTTTTGAAAA
Ppr_3           TGGGAAATTC TCTCATAGAC ATGTATATGA AATGTGGATC TGTTGAAGAG GGACTTCGGG TTTTTGAAAA
Ppr_4           TGGGAAATTC TCTCATAGAC ATGTATATGA AATGTGGATC TGTTGAAGAG GGACTTCGGG TTTTTGAAAA
Ppr_5           TGGGAAATTC TCTCATAGAC ATGTATATGA AATGTGGATC TGTTGAAGAG GGACTTCGGG TTTTTGAAAA

                ....|....| ....|....| ....|....| ....|....| ....|....| ....|....| ....|....|
                         220        230        240        250        260        270        280
Peu_1           CATGGTGGAA AAGGATCATG TATCATGGAA CACCATGATA ATAGGGTATG CMCAAAATGG CTACGGAATG
Peu_2           CATGGTGGAA AAGGATCATG TATCATGGAA CACCATGATA ATAGGGTATG CMCAAAATGG CTACGGAATG
Peu_3           CATGGTGGAA AAGGATCATG TATCATGGAA CACCATGATA ATAGGGTATG CMCAAAATGG CTACGGAATG
Peu_4           CATGGTGGAA AAGGATCATG TATCATGGAA CACCATGATA ATAGGGTATG CMCAAAATGG CTACGGAATG
Peu_5           CATGGTGGAA AAGGATCATG TATCATGGAA CACCATGATA ATAGGGTATG CMCAAAATGG CTACGGAATG
Ppr_1           CATGGTGGAA AAGGATCATG TATCATGGAA CACCATGATA ATAGGTTATG CACAAAATGG CTTCGGAATG
Ppr_2           CATGGTGGAA AAGGATCATG TATCATGGAA CACCATGATA ATAGGTTATG CACAAAATGG CTTCGGAATG
Ppr_3           CATGGTGGAA AAGGATCATG TATCATGGAA CACCATGATA ATAGGTTATG CACAAAATGG CTTCGGAATG
Ppr_4           CATGGTGGAA AAGGATCATG TATCATGGAA CACCATGATA ATAGGTTATG CACAAAATGG CTTCGGAATG
Ppr_5           CATGGTGGAA AAGGATCATG TATCATGGAA CACCATGATA ATAGGTTATG CACAAAATGG CTTCGGAATG

                ....|....| ....|....| ....|....| ....|....| ....|....| ....|....| ....|....|
                         290        300        310        320        330        340        350
Peu_1           GAGGCCCTTG AACTATTTCA GAAAATGCTG GAATCTGGAG AGAAACCAGA CCATGTTACA ATGATTGGTA
Peu_2           GAGGCCCTTG AACTATTTCA GAAAATGCTG GAATCTGGAG AGAAACCAGA CCATGTTACA ATGATTGGTA
Peu_3           GAGGCCCTTG AACTATTTCA GAAAATGCTG GAATCTGGAG AGAAACCAGA CCATGTTACA ATGATTGGTA
Peu_4           GAGGCCCTTG AACTATTTCA GAAAATGCTG GAATCTGGAG AGAAACCAGA CCATGTTACA ATGATTGGTA
Peu_5           GAGGCCCTTG AACTATTTCA GAAAATGCTG GAATCTGGAG AGAAACCAGA CCATGTTACA ATGATTGGTA
Ppr_1           GAGGCCCTTG AACTATTTCA GAAAATGCTG GAATCTGGAG AGAAACCAGA CCATGTTACA ATGATTGGTA
Ppr_2           GAGGCCCTTG AACTATTTCA GAAAATGCTG GAATCTGGAG AGAAACCAGA CCATGTTACA ATGATTGGTA
Ppr_3           GAGGCCCTTG AACTATTTCA GAAAATGCTG GAATCTGGAG AGAAACCAGA CCATGTTACA ATGATTGGTA
Ppr_4           GAGGCCCTTG AACTATTTCA GAAAATGCTG GAATCTGGAG AGAAACCAGA CCATGTTACA ATGATTGGTA
Ppr_5           GAGGCCCTTG AACTATTTCA GAAAATGCTG GAATCTGGAG AGAAACCAGA CCATGTTACA ATGATTGGTA

                ....|....| ....|....| ....|....| ....|....| ....|....| ....|....| ....|....|
                         360        370        380        390        400        410        420
```

```
Peu_1        CTCTATGTGC TTGTAGCCAC GCAGGGCTTG TTGAAGAGGG ACGRCATTAC TTTCTCTCAA TGACTAAGGA
Peu_2        CTCTATGTGC TTGTAGCCAC GCAGGGCTTG TTGAAGAGGG ACGRCATTAC TTTCTCTCAA TGACTAAGGA
Peu_3        CTCTATGTGC TTGTAGCCAC GCAGGGCTTG TTGAAGAGGG ACGRCATTAC TTTCTCTCAA TGACTAAGGA
Peu_4        CTCTATGTGC TTGTAGCCAC GCAGGGCTTG TTGAAGAGGG ACGRCATTAC TTTCTCTCAA TGACTAAGGA
Peu_5        CTCTATGTGC TTGTAGCCAC GCAGGGCTTG TTGAAGAGGG ACGRCATTAC TTTCTCTCAA TGACTAAGGA
Ppr_1        CTCTATGTGC TTGTAGCCAT GCAGGGCTTG TTGAAGAGGG ACGGCATTAC TTTTTCTCAA TGACTAAGGA
Ppr_2        CTCTATGTGC TTGTAGCCAT GCAGGGCTTG TTGAAGAGGG ACGGCATTAC TTTTTCTCAA TGACTAAGGA
Ppr_3        CTCTATGTGC TTGTAGCCAT GCAGGGCTTG TTGAAGAGGG ACGGCATTAC TTTTTCTCAA TGACTAAGGA
Ppr_4        CTCTATGTGC TTGTAGCCAT GCAGGGCTTG TTGAAGAGGG ACGGCATTAC TTTTTCTCAA TGACTAAGGA
Ppr_5        CTCTATGTGC TTGTAGCCAT GCAGGGCTTG TTGAAGAGGG ACGGCATTAC TTTTTCTCAA TGACTAAGGA

             ....|....| ....|....| ....|....| ....|....| ....|....| ....|....| ....|....|
                    430        440        450        460        470        480        490
Peu_1        ACATGGTTTG CTACCAGTGA AGGACCATTA TACATGTATG GTTGATTTAC TAGGCCGAGC TGGTTGCCTT
Peu_2        ACATGGTTTG CTACCAGTGA AGGACCATTA TACATGTATG GTTGATTTAC TAGGCCGAGC TGGTTGCCTT
Peu_3        ACATGGTTTG CTACCAGTGA AGGACCATTA TACATGTATG GTTGATTTAC TAGGCCGAGC TGGTTGCCTT
Peu_4        ACATGGTTTG CTACCAGTGA AGGACCATTA TACATGTATG GTTGATTTAC TAGGCCGAGC TGGTTGCCTT
Peu_5        ACATGGTTTG CTACCAGTGA AGGACCATTA TACATGTATG GTTGATTTAC TAGGCCGAGC TGGTTGCCTT
Ppr_1        ACATGGTTTG CTACCGGTGA AGGACCATTA TACATGTATG GTTGATTTAC TAGGCCGAGC TGGTTGCCTT
Ppr_2        ACATGGTTTG CTACCGGTGA AGGACCATTA TACATGTATG GTTGATTTAC TAGGCCGAGC TGGTTGCCTT
Ppr_3        ACATGGTTTG CTACCGGTGA AGGACCATTA TACATGTATG GTTGATTTAC TAGGCCGAGC TGGTTGCCTT
Ppr_4        ACATGGTTTG CTACCGGTGA AGGACCATTA TACATGTATG GTTGATTTAC TAGGCCGAGC TGGTTGCCTT
Ppr_5        ACATGGTTTG CTACCGGTGA AGGACCATTA TACATGTATG GTTGATTTAC TAGGCCGAGC TGGTTGCCTT

             ....|....| ....|....|
                    500        510
Peu_1        GAGGAAGCAA RGGATTTGAT
Peu_2        GAGGAAGCAA RGGATTTGAT
Peu_3        GAGGAAGCAA RGGATTTGAT
Peu_4        GAGGAAGCAA RGGATTTGAT
Peu_5        GAGGAAGCAA RGGATTTGAT
Ppr_1        GAGGAAGCAA AGGATTTGAT
Ppr_2        GAGGAAGCAA AGGATTTGAT
Ppr_3        GAGGAAGCAA AGGATTTGAT
Ppr_4        GAGGAAGCAA AGGATTTGAT
Ppr_5        GAGGAAGCAA AGGATTTGAT
```

Unigene2702_All (UDP-glucoronosyl/UDP-glucosyl transferase family protein)

```
               ....|....| ....|....| ....|....| ....|....| ....|....| ....|....| ....|....|
                        10         20         30         40         50         60         70
Peu_1          TATTCCACAT CAAATGTTGA TATTCTTCCT CTTAGTCAGG GCGAACATCC TCAAAAGAAA TCTTCCAGTG
Peu_2          TATTCCACAT CAAATGTTGA TATTCTTCCT CTTAGTCAGG GCGAACATCC TCAAAAGAAA TCTTCCAGTG
Peu_3          TATTCCACAT CAAATGTTGA TATTCTTCCT CTTAGTCAGG GCGAACATCC TCAAAAGAAA TCTTCCAGTG
Peu_4          TATTCCACAT CAAATGTTGA TATTCTTCCT CTTAGTCAGG GCGAACATCC TCAAAAGAAA TCTTCCAGTG
Peu_5          TATTCCACAT CAAATGTTGA TATTCTTCCT CTTAGTCAGG GCGAACATCC TCAAAAGAAA TCTTCCAGTG
Ppr_1          TATTCCACAT CAAATGTTGA TATTCTTCCT CTTAGTCAGG GCGAACATCC TCAAAAGAAA TCTTCCAGTG
Ppr_2          TATTCCACAT CAAATGTTGA TATTCTTCCT CTTAGTCAGG GCGAACATCC TCAAAAGAAA TCTTCCAGTG
Ppr_3          TATTCCACAT CAAATGTTGA TATTCTTCCT CTTAGTCAGG GCGAACATCC TCAAAAGAAA TCTTCCAGTG
Ppr_4          TATTCCACAT CAAATGTTGA TATTCTTCCT CTTAGTCAGG GCGAACATCC TCAAAAGAAA TCTTCCAGTG

               ....|....| ....|....| ....|....| ....|....| ....|....| ....|....| ....|....|
                        80         90        100        110        120        130        140
Peu_1          GACAGAGCAG TGCAGATGAA TTGTATCCAT CTGATCATGC TGCAGCCTTC CTTTCCCTAA TGAAAAGCAG
Peu_2          GACAGAGCAG TGCAGATGAA TTGTATCCAT CTGATCATGC TGCAGCCTTC CTTTCCCTAA TGAAAAGCAG
Peu_3          GACAGAGCAG TGCAGATGAA TTGTATCCAT CTGATCATGC TGCAGCCTTC CTTTCCCTAA TGAAAAGCAG
Peu_4          GACAGAGCAG TGCAGATGAA TTGTATCCAT CTGATCATGC TGCAGCCTTC CTTTCCCTAA TGAAAAGCAG
Peu_5          GACAGAGCAG TGCAGATGAA TTGTATCCAT CTGATCATGC TGCAGCCTTC CTTTCCCTAA TGAAAAGCAG
Ppr_1          GACAGAGCAG TGCAGATGAA TTGTATCCAT CTGATCATGC TGCAGCCTTC CTTTCCCTAA TGAAAAGCAG
Ppr_2          GACAGAGCAG TGCAGATGAA TTGTATCCAT CTGATCATGC TGCAGCCTTC CTTTCCCTAA TGAAAAGCAG
Ppr_3          GACAGAGCAG TGCAGATGAA TTGTATCCAT CTGATCATGC TGCAGCCTTC CTTTCCCTAA TGAAAAGCAG
Ppr_4          GACAGAGCAG TGCAGATGAA TTGTATCCAT CTGATCATGC TGCAGCCTTC CTTTCCCTAA TGAAAAGCAG

               ....|....| ....|....| ....|....| ....|....| ....|....| ....|....| ....|....|
                       150        160        170        180        190        200        210
Peu_1          CGGAACACTG GATGTATTAT TATCACAGGG AAAGGAGTAC GTACATGTGG TGAGCTCCGA TAATGTAGCT
Peu_2          CGGAACACTG GATGTATTAT TATCACAGGG AAAGGAGTAC GTACATGTGG TGAGCTCCGA TAATGTAGCT
Peu_3          CGGAACACTG GATGTATTAT TATCACAGGG AAAGGAGTAC GTACATGTGG TGAGCTCCGA TAATGTAGCT
Peu_4          CGGAACACTG GATGTATTAT TATCACAGGG AAAGGAGTAC GTACATGTGG TGAGCTCCGA TAATGTAGCT
Peu_5          CGGAACACTG GATGTATTAT TATCACAGGG AAAGGAGTAC GTACATGTGG TGAGCTCCGA TAATGTAGCT
Ppr_1          CGGAACACTG GATGTATTAT TATCACAGGG AAAGGAGTAC GTACATGTGG TGAGCTCCGA TAATGTAGCT
Ppr_2          CGGAACACTG GATGTATTAT TATCACAGGG AAAGGAGTAC GTACATGTGT TGAGCTCTGA TAATGTAGCT
Ppr_3          CGGAACACTG GATGTATTAT TATCACAGGG AAAGGAGTAC GTACATGTGG TGAGCTCTGA TAATGTAGCT
Ppr_4          CGGAACACTG GATGTATTAT TATCACAGGG AAAGGAGTAC GTACATGTGT TGAGCTCTGA TAATGTAGCT

               ....|....| ....|....| ....|....| ....|....| ....|....| ....|....| ....|....|
                       220        230        240        250        260        270        280
Peu_1          GCAGCTGTTG ATCCAAGAAT CATGAGTCAT TTGAGCCAAA ACAATATTGA ATACTGTATG GAGGTGACAC
Peu_2          GCAGCTGTTG ATCCAAGAAT CATGAGTCAT TTGAGCCAAA ACAATATTGA ATACTGTATG GAGGTGACAC
Peu_3          GCAGCTGTTG ATCCAAGAAT CATGAGTCAT TTGAGCCAAA ACAATATTGA ATACTGTATG GAGGTGACAC
Peu_4          GCAGCTGTTG ATCCAAGAAT CATGAGTCAT TTGAGCCAAA ACAATATTGA ATACTGTATG GAGGTGACAC
Peu_5          GCAGCTGTTG ATCCAAGAAT CATGAGTCAT TTGAGCCAAA ACAATATTGA ATACTGTATG GAGGTGACAC
Ppr_1          GCAGCTGTTG ATCCAAGAAT CATGAGTCAT TTGAGCCAAA ACAATATTGA ATACTGTATG GAGGTGACAC
Ppr_2          GCAGCTGTTG ATCCAAGAAT CATGAGTCAT TTGAGCCAAA ACAATATTGA ATACTGTATG GAGGTGACAC
Ppr_3          GCAGCTGTTG ATCCAAGAAT CATGAGTCAT TTGAGCCAAA ACAATATTGA ATACTGTATG GAGGTGACAC
Ppr_4          GCAGCTGTTG ATCCAAGAAT CATGAGTCAT TTGAGCCAAA ACAATATTGA ATACTGTATG GAGGTGACAC

               ....|....| ....|....| ....|....| ....|....| ....|....| ....|....| ....|....|
                       290        300        310        320        330        340        350
Peu_1          CAACCACTTC ATATCTTTCA AAAAGTAAAA TGGCCAGTCA GCAGCAAGGA AAGTTTGAGC TTGCAGAAAT
Peu_2          CAACCACTTC ATATCTTTCA AAAAGTAAAA TGGCCAGTCA GCAGCAAGGA AAGTTTGAGC TTGCAGAAAT
Peu_3          CAACCACTTC ATATCTTTCA AAAAGTAAAA TGGCCAGTCA GCAGCAAGGA AAGTTTGAGC TTGCAGAAAT
Peu_4          CAACCACTTC ATATCTTTCA AAAAGTAAAA TGGCCAGTCA GCAGCAAGGA AAGTTTGAGC TTGCAGAAAT
Peu_5          CAACCACTTC ATATCTTTCA AAAAGTAAAA TGGCCAGTCA GCAGCAAGGA AAGTTTGAGC TTGCAGAAAT
Ppr_1          CAACCACTTC ATATCTTTCA AAAAGTAAAA TGGCCAATCA GCAGCAAGGA ATGTTTGAGC TTGCAGAAAT
Ppr_2          CAACCACTTC ATATCTTTCA AAAAGTAAAA TGGCCAATCA GCAGCAAGGA ATGTTTGAGC TTGCAGAAAT
Ppr_3          CAACCACTTC ATATCTTTCA AAAAGTAAAA TGGCCAATCA GCAGCAAGGA ATGTTTGAGC TTGCAGAAAT
Ppr_4          CAACCACTTC ATATCTTTCA AAAAGTAAAA TGGCCAATCA GCAGCAAGGA ATGTTTGAGC TTGCAGAAAT

               ....|....| ....|....| ....|....| ....|....| ....|....| ....|....| ....|....|
                       360        370        380        390        400        410        420
Peu_1          AGCGCGAGCA CTTCCTAAAG ATTCAACGGA GAAGTTCAAA TTTATCGACA CAAGAAGTTT GTGGGTGAAT
Peu_2          AGCGCGAGCA CTTCCTAAAG ATTCAACGGA GAAGTTCAAA TTTATCGACA CAAGAAGTTT GTGGGTGAAT
Peu_3          AGCGCGAGCA CTTCCTAAAG ATTCAACGGA GAAGTTCAAA TTTATCGACA CAAGAAGTTT GTGGGTGAAT
Peu_4          AGCGCGAGCA CTTCCTAAAG ATTCAACGGA GAAGTTCAAA TTTATCGACA CAAGAAGTTT GTGGGTGAAT
Peu_5          AGCGCGAGCA CTTCCTAAAG ATTCAACGGA GAAGTTCAAA TTTATCGACA CAAGAAGTTT GTGGGTGAAT
```

```
Ppr_1          AGCGCGAACA CTTCCTAAAG ATTCAACGGA GAAGTTCAAA TTTATCGACA CAAGAAGTTT GTGGGTGAAT
Ppr_2          AGCGCGAACA CTTCCTAAAG ATTCAACGGA GAAGTTCAAA TTTATCGACA CAAGAAGTTT GTGGGTGAAT
Ppr_3          AGCGCGAACA CTTCCTAAAG ATTCAACGGA GAAGTTCAAA TTTATCGACA CAAGAAGTTT GTGGGTGAAT
Ppr_4          AGCGCGAACA CTTCCTAAAG ATTCAACGGA GAAGTTCAAA TTTATCGACA CAAGAAGTTT GTGGGTGAAT

               ....|....| ....|....| ....|....| ....|....| ....|....| ....|....| ....|....|
                        430        440        450        460        470        480        490
Peu_1          TTGAAAGCAA TCAGAAGGCT TGTTGATACC AATGCCCTGA AGATAGAGAA CCTGTCTATT TCAAAGGAAG
Peu_2          TTGAAAGCAA TCAGAAGGCT TGTTGATACC AATGCCCTGA AGATAGAGAA CCTGTCTATT TCAAAGGAAG
Peu_3          TTGAAAGCAA TCAGAAGGCT TGTTGATACC AATGCCCTGA AGATAGAGAA CCTGTCTATT TCAAAGGAAG
Peu_4          TTGAAAGCAA TCAGAAGGCT TGTTGATACC AATGCCCTGA AGATAGAGAA CCTGTCTATT TCAAAGGAAG
Peu_5          TTGAAAGCAA TCAGAAGGCT TGTTGATACC AATGCCCTGA AGATAGAGAA CCTGTCTATT TCAAAGGAAG
Ppr_1          TTGAAAGCAA TCAGAAGGCT TGTTGATACC AATGCCCTGA AGATAGAGAA CCTGTCTATT TCAAAGGAAG
Ppr_2          TTGAAAGCAA TCAGAAGGCT TGTTGATACC AATGCCCTGA AGATAGAGAA CCTGTCTATT TCAAAGGAAG
Ppr_3          TTGAAAGCAA TCAGAAGGCT TGTTGATACC AATGCCCTGA AGATAGAGAA CCTGTCTATT TCAAAGGAAG
Ppr_4          TTGAAAGCAA TCAGAAGGCT TGTTGATACC AATGCCCTGA AGATAGAGAA CCTGTCTATT TCAAAGGAAG

               ....|....| ....|....| ....|....| ....|....| ....|....| ....|....| ....|....|
                        500        510        520        530        540        550        560
Peu_1          TGGAAGGTGA TCAAATGGTT TTGCAAGAGA CAGCAGCCGG TTCGACAATA CAGTTATTTG ATAAAGCCAT
Peu_2          TGGAAGGTGA TCAAATGGTT TTGCAAGAGA CAGCAGCCGG TTCGACAATA CAGTTATTTG ATAAAGCCAT
Peu_3          TGGAAGGTGA TCAAATGGTT TTGCAAGAGA CAGCAGCCGG TTCGACAATA CAGTTATTTG ATAAAGCCAT
Peu_4          TGGAAGGTGA TCAAATGGTT TTGCAAGAGA CAGCAGCCGG TTCGACAATA CAGTTATTTG ATAAAGCCAT
Peu_5          TGGAAGGTGA TCAAATGGTT TTGCAAGAGA CAGCAGCCGG TTCGACAATA CAGTTATTTG ATAAAGCCAT
Ppr_1          TGGAAGGTGA TCAAATGGTT TTGCAAGAGA CAGTAGCCGG TTCGACAATA CAGTTATTTG ATAAAGCCAT
Ppr_2          TGGAAGGTGA TCAAATGGTT TTGCAAGAGA CAGTAGCCGG TTCGACAATA CAGTTATTTG ATAAAGCCAT
Ppr_3          TGGAAGGTGA TCAAATGGTT TTGCAAGAGA CAGTAGCCGG TTCGACAATA CAGTTATTTG ATAAAGCCAT
Ppr_4          TGGAAGGTGA TCAAATGGTT TTGCAAGAGA CAGTAGCCGG TTCGACAATA CAGTTATTTG ATAAAGCCAT

               ....|....| ....|....| ....|....| ....|....| ....|....| ....|....| ....|....|
                        570        580        590        600        610        620        630
Peu_1          CGGCGTTAAT GTCCCTCAAT TTCGGGTTGT GCAACTAAAT GCAACATCAG ACTTGCTTCT TCTTCAGTCA
Peu_2          CGGCGTTAAT GTCCCTCAAT TTCGGGTTGT GCAACTAAAT GCAACATCAG ACTTGCTTCT TCTTCAGTCA
Peu_3          CGGCGTTAAT GTCCCTCAAT TTCGGGTTGT GCAACTAAAT GCAACATCAG ACTTGCTTCT TCTTCAGTCA
Peu_4          CGGCGTTAAT GTCCCTCAAT TTCGGGTTGT GCAACTAAAT GCAACATCAG ACTTGCTTCT TCTTCAGTCA
Peu_5          CGGCGTTAAT GTCCCTCAAT TTCGGGTTGT GCAACTAAAT GCAACATCAG ACTTGCTTCT TCTTCAGTCA
Ppr_1          TGGCGTTAAT GTCCCTCAAT TTCGGGTTGT GCAACTAAAT GCAACATCAG ACTTGCTTCT TCTTCAGTCA
Ppr_2          TGGCGTTAAT GTCCCTCAAT TTCGGGTTGT GCAACTAAAT GCAACATCAG ACTTGCTTCT TCTTCAGTCA
Ppr_3          TGGCGTTAAT GTCCCTCAAT TTCGGGTTGT GCAACTAAAT GCAACATCAG ACTTGCTTCT TCTTCAGTCA
Ppr_4          TGGCGTTAAT GTCCCTCAAT TTCGGGTTGT GCAACTAAAT GCAACATCAG ACTTGCTTCT TCTTCAGTCA

               ....|....| ....|....| ....|....| ....|....| ....|....| ....|....| ....|....|
                        640        650        660        670        680        690        700
Peu_1          GATCTGTACT CCACCTCTGA AGGTGTTTTA GTTCGGAACA CAGCTCAAGC TAACCCTGCA AATCCATGTA
Peu_2          GATCTGTACT CCACCTCTGA AGGTGTTTTA GTTCGGAACA CAGCTCAAGC TAACCCTGCA AATCCATGTA
Peu_3          GATCTGTACT CCACCTCTGA AGGTGTTTTA GTTCGGAACA CAGCTCAAGC TAACCCTGCA AATCCATGTA
Peu_4          GATCTGTACT CCACCTCTGA AGGTGTTTTA GTTCGGAACA CAGCTCAAGC TAACCCTGCA AATCCATGTA
Peu_5          GATCTGTACT CCACCTCTGA AGGTGTTTTA GTTCGGAACA CAGCTCAAGC TAACCCTGCA AATCCATGTA
Ppr_1          GATCTGTACT CCACCTCTGA AGGTGTTTTA GTTCGGAACA CAGCTCAAGC TAACCCTGCA AATCCATGTA
Ppr_2          GATCTGTACT CCACCTCTGA AGGTGTTTTA GTTCGGAACA CAGCTCAAGC TAACCCTGCA AATCCATGTA
Ppr_3          GATCTGTACT CCACCTCTGA AGGTGTTTTA GTTCGGAACA CAGCTCAAGC TAACCCTGCA AATCCATGTA
Ppr_4          GATCTGTACT CCACCTCTGA AGGTGTTTTA GTTCGGAACA CAGCTCAAGC TAACCCTGCA AATCCATGTA

               ...

Peu_1          TTG
Peu_2          TTG
Peu_3          TTG
Peu_4          TTG
Peu_5          TTG
Ppr_1          TTG
Ppr_2          TTG
Ppr_3          TTG
Ppr_4          TTG
```

Unigene37561_All (heat stress transcription factor A3)

```
               ....|....| ....|....| ....|....| ....|....| ....|....| ....|....| ....|....|
                      10         20         30         40         50         60         70
Peu_1          GATGGCCATC TCATATTGAT TTTACTGGGG AAATGTTGGG GGCAAAGAAG CTTAGGAAGA CTATGTCACA
Peu_2          GATGGCCATC TCATATTGAT TTTACTGGGG AAATGTTGGG GGCAAAGAAG CTTAGGAAGA CTATGTCACA
Peu_3          GATGGCCATC TCATATTGAT TTTACTGGGG AAATGTTGGG GGCAAAGAAG CTTAGGAAGA CTATGTCACA
Peu_4          GATGGCCATC TCATATTGAT TTTACTGGGG AAATGTTGGG GGCAAAGAAG CTTAGGAAGA CTATGTCACA
Peu_5          GATGGCCATC TCATATTGAT TTTACTGGGG AAATGTTGGG GGCAAAGAAG CTTAGGAAGA CTATGTCACA
Ppr_1          GATGGCCATC TCATATTGAT TTTACTGGGG AAATGTTGGG GGCAAAGAAG CTTAGGAAGA CTATGTCACA
Ppr_2          GATGGCCATC TCATATTGAT TTTACTGGGG AAATGTTGGG GGCAAAGAAG CTTAGGAAGA CTATGTCACA
Ppr_3          GATGGCCATC TCATATTGAT TTTACTGGGG AAATGTTGGG GGCAAAGAAG CTTAGGAAGA CTATGTCACA
Ppr_4          GATGGCCATC TCATATTGAT TTTACTGGGG AAATGTTGGG GGCAAAGAAG CTTAGGAAGA CTATGTCACA
Ppr_5          GATGGCCATC TCATATTGAT TTTACTGGGG AAATGTTGGG GGCAAAGAAG CTTAGGAAGA CTATGTCACA

               ....|....| ....|....| ....|....| ....|....| ....|....| ....|....| ....|....|
                      80         90        100        110        120        130        140
Peu_1          TCCAGCTAAT GGTAGCGCTG ATGATCTAGT TTTTGGAAAT GAAGTTAGAC CTCGTAAGGA ATTTTTCCGA
Peu_2          TCCAGCTAAT GGTAGCGCTG ATGATCTAGT TTTTGGAAAT GAAGTTAGAC CTCGTAAGGA ATTTTTCCGA
Peu_3          TCCAGCTAAT GGTAGCGCTG ATGATCTAGT TTTTGGAAAT GAAGTTAGAC CTCGTAAGGA ATTTTTCCGA
Peu_4          TCCAGCTAAT GGTAGCGCTG ATGATCTAGT TTTTGGAAAT GAAGTTAGAC CTCGTAAGGA ATTTTTCCGA
Peu_5          TCCAGCTAAT GGTAGCGCTG ATGATCTAGT TTTTGGAAAT GAAGTTAGAC CTCGTAAGGA ATTTTTCCGA
Ppr_1          TCCAGCTAAT GGTAGCGCTG ATGATCTAGT TTTTGGAAAT GAAGTTAGAC CTCGTAAGGA ATTTTTCCGA
Ppr_2          TCCAGCTAAT GGTAGCGCTG ATGATCTAGT TTTTGGAAAT GAAGTTAGAC CTCGTAAGGA ATTTTTCCGA
Ppr_3          TCCAGCTAAT GGTAGCGCTG ATGATCTAGT TTTTGGAAAT GAAGTTAGAC CTCGTAAGGA ATTTTTCCGA
Ppr_4          TCCAGCTAAT GGTAGCGCTG ATGATCTAGT TTTTGGAAAT GAAGTTAGAC CTCGTAAGGA ATTTTTCCGA
Ppr_5          TCCAGCTAAT GGTAGCGCTG ATGATCTAGT TTTTGGAAAT GAAGTTAGAC CTCGTAAGGA ATTTTTCCGA

               ....|....| ....|....| ....|....| ....|....| ....|....| ....|....| ....|....|
                     150        160        170        180        190        200        210
Peu_1          AATGACTCTC TTCCTAATGA GACGTTTGTA ACTATTTCTC ACGTCAGTCT TAAAACTCAC CCCTCTCAGT
Peu_2          AATGACTCTC TTCCTAATGA GACGTTTGTA ACTATTTCTC ACGTCAGTCT TAAAACTCAC CCCTCTCAGT
Peu_3          AATGACTCTC TTCCTAATGA GACGTTTGTA ACTATTTCTC ACGTCAGTCT TAAAACTCAC CCCTCTCAGT
Peu_4          AATGACTCTC TTCCTAATGA GACGTTTGTA ACTATTTCTC ACGTCAGTCT TAAAACTCAC CCCTCTCAGT
Peu_5          AATGACTCTC TTCCTAATGA GACGTTTGTA ACTATTTCTC ACGTCAGTCT TAAAACTCAC CCCTCTCAGT
Ppr_1          AATGACTCTC TTCCTAATGA GACGTTTGTA ACTATTTCTC ACGTCAGTCT TAAAACTCAC CCCTCTCAGT
Ppr_2          AATGACTCTC TTCCTAATGA GACGTTTGTA ACTATTTCTC ACGTCAGTCT TAAAACTCAC CCCTCTCAGT
Ppr_3          AATGACTCTC TTCCTAATGA GACGTTTGTA ACTATTTCTC ACGTCAGTCT TAAAACTCAC CCCTCTCAGT
Ppr_4          AATGACTCTC TTCCTAATGA GACGTTTGTA ACTATTTCTC ACGTCAGTCT TAAAACTCAC CCCTCTCAGT
Ppr_5          AATGACTCTC TTCCTAATGA GACGTTTGTA ACTATTTCTC ACGTCAGTCT TAAAACTCAC CCCTCTCAGT

               ....|....| ....|....| ....|....| ....|....| ....|....| ....|....| ....|....|
                     220        230        240        250        260        270        280
Peu_1          TGCCACCACC TTCTAGACCA CCGCCTGCAT TAGATGTCAA CAAGAGGGAT TCTTGTAAAT CAACTCCAAA
Peu_2          TGCCACCACC TTCTAGACCA CCGCCTGCAT TAGATGTCAA CAAGAGGGAT TCTTGTAAAT CAACTCCAAA
Peu_3          TGCCACCACC TTCTAGACCA CCGCCTGCAT TAGATGTCAA CAAGAGGGAT TCTTGTAAAT CAACTCCAAA
Peu_4          TGCCACCACC TTCTAGACCA CCGCCTGCAT TAGATGTCAA CAAGAGGGAT TCTTGTAAAT CAACTCCAAA
Peu_5          TGCCACCACC TTCTAGACCA CCGCCTGCAT TAGATGTCAA CAAGAGGGAT TCTTGTAAAT CAACTCCAAA
Ppr_1          TGCCACCACC TTCTAGACCA CCGCCTGCAT TAGATGTCAA CAAGAGGGAT TCTTGTAAAT CAACTCCAAA
Ppr_2          TGCCACCACC TTCTAGACCA CCGCCTGCAT TAGATGTCAA CAAGAGGGAT TCTTGTAAAT CAACTCCAAA
Ppr_3          TGCCACCACC TTCTAGACCA CCGCCTGCAT TAGATGTCAA CAAGAGGGAT TCTTGTAAAT CAACTCCAAA
Ppr_4          TGCCACCACC TTCTAGACCA CCGCCTGCAT TAGATGTCAA CAAGAGGGAT TCTTGTAAAT CAACTCCAAA
Ppr_5          TGCCACCACC TTCTAGACCA CCGCCTGCAT TAGATGTCAA CAAGAGGGAT TCTTGTAAAT CAACTCCAAA

               ....|....| ....|....| ....|....| ....|....| ....|....| ....|....| ....|....|
                     290        300        310        320        330        340        350
Peu_1          CTGTCACAGC GCTGCTTCCT CAGGAAGTGC TGGTGATAGT TCTCCACCTT ACTTTGATGT TGAGGTAGAT
Peu_2          CTGTCACAGC GCTGCTTCCT CAGGAAGTGC TGGTGATAGT TCTCCACCTT ACTTTGATGT TGAGGTAGAT
Peu_3          CTGTCACAGC GCTGCTTCCT CAGGAAGTGC TGGTGATAGT TCTCCACCTT ACTTTGATGT TGAGGTAGAT
Peu_4          CTGTCACAGC GCTGCTTCCT CAGGAAGTGC TGGTGATAGT TCTCCACCTT ACTTTGATGT TGAGGTAGAT
Peu_5          CTGTCACAGC GCTGCTTCCT CAGGAAGTGC TGGTGATAGT TCTCCACCTT ACTTTGATGT TGAGGTAGAT
Ppr_1          CTGTCACAGC GCTGCTTCCT CAGGAAGTGC TGGTGATAGT TCTCCACCTT ACTTTGATGT TGAGGTAGAT
Ppr_2          CTGTCACAGC GCTGCTTCCT CAGGAAGTGC TGGTGATAGT TCTCCACCTT ACTTTGATGT TGAGGTAGAT
Ppr_3          CTGTCACAGC GCTGCTTCCT CAGGAAGTGC TGGTGATAGT TCTCCACCTT ACTTTGATGT TGAGGTAGAT
Ppr_4          CTGTCACAGC GCTGCTTCCT CAGGAAGTGC TGGTGATAGT TCTCCACCTT ACTTTGATGT TGAGGTAGAT
Ppr_5          CTGTCACAGC GCTGCTTCCT CAGGAAGTGC TGGTGATAGT TCTCCACCTT ACTTTGATGT TGAGGTAGAT

               ....|....| ....|....| ....|....| ....|....| ....|....| ....|....| ....|....|
                     360        370        380        390        400        410        420
```

```
Peu_1        GCAAGTTCCT CTGCTGCTGC CTCTGCTGCT GCTATAAAAG AAGCTATGGA GAAAGCTCAA GTCAAGCTGA
Peu_2        GCAAGTTCCT CTGCTGCTGC CTCTGCTGCT GCTATAAAAG AAGCTATGGA GAAAGCTCAA GTCAAGCTGA
Peu_3        GCAAGTTCCT CTGCTGCTGC CTCTGCTGCT GCTATAAAAG AAGCTATGGA GAAAGCTCAA GTCAAGCTGA
Peu_4        GCAAGTTCCT CTGCTGCTGC CTCTGCTGCT GCTATAAAAG AAGCTATGGA GAAAGCTCAA GTCAAGCTGA
Peu_5        GCAAGTTCCT CTGCTGCTGC CTCTGCTGCT GCTATAAAAG AAGCTATGGA GAAAGCTCAA GTCAAGCTGA
Ppr_1        GCAAGTTCCT CTGCTGCTGC CTCTGCTGCT GCTATAAAAG AAGCTATGGA GAAAGCTCAA GTCAAGCTGA
Ppr_2        GCAAGTTCCT CTGCTGCTGC CTCTGCTGCT GCTATAAAAG AAGCTATGGA GAAAGCTCAA GTCAAGCTGA
Ppr_3        GCAAGTTCCT CTGCTGCTGC CTCTGCTGCT GCTATAAAAG AAGCTATGGA GAAAGCTCAA GTCAAGCTGA
Ppr_4        GCAAGTTCCT CTGCTGCTGC CTCTGCTGCT GCTATAAAAG AAGCTATGGA GAAAGCTCAA GTCAAGCTGA
Ppr_5        GCAAGTTCCT CTGCTGCTGC CTCTGCTGCT GCTATAAAAG AAGCTATGGA GAAAGCTCAA GTCAAGCTGA

             ....|....| ....|....| ....|....| ....|....| ....|....| ....|....| ....|....|
                    430        440        450        460        470        480        490
Peu_1        AAAGTGCAAA AGAATTGATG GACAGGAAGA GGGGTGGTTT TCAAAACCAT ACAAAATCGG CTTCAAAAAA
Peu_2        AAAGTGCAAA AGAATTGATG GACAGGAAGA GGGGTGGTTT TCAAAACCAT ACAAAATCGG CTTCAAAAAA
Peu_3        AAAGTGCAAA AGAATTGATG GACAGGAAGA GGGGTGGTTT TCAAAACCAT ACAAAATCGG CTTCAAAAAA
Peu_4        AAAGTGCAAA AGAATTGATG GACAGGAAGA GGGGTGGTTT TCAAAACCAT ACAAAATCGG CTTCAAAAAA
Peu_5        AAAGTGCAAA AGAATTGATG GACAGGAAGA GGGGTGGTTT TCAAAACCAT ACAAAATCGG CTTCAAAAAA
Ppr_1        AAAGTGCAAA AGAATTGATG GACAGGAAGA GGGGTGGTTT TCAAAACCAT ACAAAATCGG CTTCAAAAAA
Ppr_2        AAAGTGCAAA AGAATTGATG GACAGGAAGA GGGGTGGTTT TCAAAACCAT ACAAAATCGG CTTCAAAAAA
Ppr_3        AAAGTGCAAA AGAATTGATG GACAGGAAGA GGGGTGGTTT TCAAAACCAT ACAAAATCGG CTTCAAAAAA
Ppr_4        AAAGTGCAAA AGAATTGATG GACAGGAAGA GGGGTGGTTT TCAAAACCAT ACAAAATCGG CTTCAAAAAA
Ppr_5        AAAGTGCAAA AGAATTGATG GACAGGAAGA GGGGTGGTTT TCAAAACCAT ACAAAATCGG CTTCAAAAAA

             ....|....| ....|....| ....|....| ....|....| ....|....| ....|....| ....|....|
                    500        510        520        530        540        550        560
Peu_1        TGACAAAAAG GATAGAGAAG GTAGGGTGGT TAAGATTGTT GATGTGTCTG GTAGTACAAA ATATGAAGGG
Peu_2        TGACAAAAAG GATAGAGAAG GTAGGGTGGT TAAGATTGTT GATGTGTCTG GTAGTACAAA ATATGAAGGG
Peu_3        TGACAAAAAG GATAGAGAAG GTAGGGTGGT TAAGATTGTT GATGTGTCTG GTAGTACAAA ATATGAAGGG
Peu_4        TGACAAAAAG GATAGAGAAG GTAGGGTGGT TAAGATTGTT GATGTGTCTG GTAGTACAAA ATATGAAGGG
Peu_5        TGACAAAAAG GATAGAGAAG GTAGGGTGGT TAAGATTGTT GATGTGTCTG GTAGTACAAA ATATGAAGGG
Ppr_1        TGACAAAAAG GATAGAGAAG GTAGGGTGGT TAAGATTGTT GATGTGTCTG GTAGTACAAA ATATGAAGGG
Ppr_2        TGACAAAAAG GATAGAGAAG GTAGGGTGGT TAAGATTGTT GATGTGTCTG GTAGTACAAA ATATGAAGGG
Ppr_3        TGACAAAAAG GATAGAGAAG GTAGGGTGGT TAAGATTGTT GATGTGTCTG GTAGTACAAA ATATGAAGGG
Ppr_4        TGACAAAAAG GATAGAGAAG GTAGGGTGGT TAAGATTGTT GATGTGTCTG GTAGTACAAA ATATGAAGGG
Ppr_5        TGACAAAAAG GATAGAGAAG GTAGGGTGGT TAAGATTGTT GATGTGTCTG GTAGTACAAA ATATGAAGGG

             ....|....| ....|....| ....|....| ....|....| ....|....| ....|....| ....|....|
                    570        580        590        600        610        620        630
Peu_1        GTGCAAGGCA CTTGTGAAAG TGAAGAGAAT GGAATGGATG ACAGGCWAAA GGTTAAAATT CCAGATTCTT
Peu_2        GTGCAAGGCA CTTGTGAAAG TGAAGAGAAT GGAATGGATG ACAGGCWAAA GGTTAAAATT CCAGATTCTT
Peu_3        GTGCAAGGCA CTTGTGAAAG TGAAGAGAAT GGAATGGATG ACAGGCWAAA GGTTAAAATT CCAGATTCTT
Peu_4        GTGCAAGGCA CTTGTGAAAG TGAAGAGAAT GGAATGGATG ACAGGCWAAA GGTTAAAATT CCAGATTCTT
Peu_5        GTGCAAGGCA CTTGTGAAAG TGAAGAGAAT GGAATGGATG ACAGGCWAAA GGTTAAAATT CCAGATTCTT
Ppr_1        GTGCAAGGCA CTTGTGAAAG TGAAGAGAAT GGAATGGATG ACAGGCAAAA GGTTAAAATT CCAGATTCTT
Ppr_2        GTGCAAGGCA CTTGTGAAAG TGAAGAGAAT GGAATGGATG ACAGGCAAAA GGTTAAAATT CCAGATTCTT
Ppr_3        GTGCAAGGCA CTTGTGAAAG TGAAGAGAAT GGAATGGATG ACAGGCAAAA GGTTAAAATT CCAGATTCTT
Ppr_4        GTGCAAGGCA CTTGTGAAAG TGAAGAGAAT GGAATGGATG ACAGGCAAAA GGTTAAAATT CCAGATTCTT
Ppr_5        GTGCAAGGCA CTTGTGAAAG TGAAGAGAAT GGAATGGATG ACAGGCAAAA GGTTAAAATT CCAGATTCTT

             ....|....| ....|....| ....|....| ....|....| ....|....| ....|....| ....|....|
                    640        650        660        670        680        690        700
Peu_1        TAGAAGGGAA AAGGCATCAA AATGCAGCAA AAAAGTCTTC TGATGAGAAG CATGGAAGGG AATCCTTGTC
Peu_2        TAGAAGGGAA AAGGCATCAA AATGCAGCAA AAAAGTCTTC TGATGAGAAG CATGGAAGGG AATCCTTGTC
Peu_3        TAGAAGGGAA AAGGCATCAA AATGCAGCAA AAAAGTCTTC TGATGAGAAG CATGGAAGGG AATCCTTGTC
Peu_4        TAGAAGGGAA AAGGCATCAA AATGCAGCAA AAAAGTCTTC TGATGAGAAG CATGGAAGGG AATCCTTGTC
Peu_5        TAGAAGGGAA AAGGCATCAA AATGCAGCAA AAAAGTCTTC TGATGAGAAG CATGGAAGGG AATCCTTGTC
Ppr_1        TAGAAGGGAA AAGGCATCAA AATGCAGCAA AAAAGTCTTC TGATGAGAAG CATGGAAGGG AATCCTTGTC
Ppr_2        TAGAAGGGAA AAGGCATCAA AATGCAGCAA AAAAGTCTTC TGATGAGAAG CATGGAAGGG AATCCTTGTC
Ppr_3        TAGAAGGGAA AAGGCATCAA AATGCAGCAA AAAAGTCTTC TGATGAGAAG CATGGAAGGG AATCCTTGTC
Ppr_4        TAGAAGGGAA AAGGCATCAA AATGCAGCAA AAAAGTCTTC TGATGAGAAG CATGGAAGGG AATCCTTGTC
Ppr_5        TAGAAGGGAA AAGGCATCAA AATGCAGCAA AAAAGTCTTC TGATGAGAAG CATGGAAGGG AATCCTTGTC

             ....|....| ....|....| ....|....| ....|....| ....|....| ....|....| ....|....|
                    710        720        730        740        750        760        770
Peu_1        ATCTCAAGGA TCTGATAAAA TTGATGAAGC TAGTGAATGG AAAGAAGCTA CTCAATTTTT TGAACTGGTG
Peu_2        ATCTCAAGGA TCTGATAAAA TTGATGAAGC TAGTGAATGG AAAGAAGCTA CTCAATTTTT TGAACTGGTG
Peu_3        ATCTCAAGGA TCTGATAAAA TTGATGAAGC TAGTGAATGG AAAGAAGCTA CTCAATTTTT TGAACTGGTG
Peu_4        ATCTCAAGGA TCTGATAAAA TTGATGAAGC TAGTGAATGG AAAGAAGCTA CTCAATTTTT TGAACTGGTG
```

```
Peu_5         ATCTCAAGGA TCTGATAAAA TTGATGAAGC TAGTGAATGG AAAGAAGCTA CTCAATTTTT TGAACTGGTG
Ppr_1         ATCTCAAGGA TCTGATAAAA TTGATGAAGC TAGTGARTGG AAAGAAGCTA CTCAATTTTT TGAACTGGTG
Ppr_2         ATCTCAAGGA TCTGATAAAA TTGATGAAGC TAGTGARTGG AAAGAAGCTA CTCAATTTTT TGAACTGGTG
Ppr_3         ATCTCAAGGA TCTGATAAAA TTGATGAAGC TAGTGARTGG AAAGAAGCTA CTCAATTTTT TGAACTGGTG
Ppr_4         ATCTCAAGGA TCTGATAAAA TTGATGAAGC TAGTGARTGG AAAGAAGCTA CTCAATTTTT TGAACTGGTG
Ppr_5         ATCTCAAGGA TCTGATAAAA TTGATGAAGC TAGTGARTGG AAAGAAGCTA CTCAATTTTT TGAACTGGTG

              ....|....| ....|....| ....|....| ....|....| ....|....| ....|....| ....|....|
                      780        790        800        810        820        830        840
Peu_1         AGAACAAATG TACCCAGGAA AGTCGTTGAC TTGTCAAATA ATGAAAACAT TTTTCTGCAG AACACAAATA
Peu_2         AGAACAAATG TACCCAGGAA AGTCGTTGAC TTGTCAAATA ATGAAAACAT TTTTCTGCAG AACACAAATA
Peu_3         AGAACAAATG TACCCAGGAA AGTCGTTGAC TTGTCAAATA ATGAAAACAT TTTTCTGCAG AACACAAATA
Peu_4         AGAACAAATG TACCCAGGAA AGTCGTTGAC TTGTCAAATA ATGAAAACAT TTTTCTGCAG AACACAAATA
Peu_5         AGAACAAATG TACCCAGGAA AGTCGTTGAC TTGTCAAATA ATGAAAACAT TTTTCTGCAG AACACAAATA
Ppr_1         AGAACAAATG TACCCAGGAA AGTCGTTGAC TTGTCAAATA ATGAAAACAT TTTTCTGCAG AACACAAATA
Ppr_2         AGAACAAATG TACCCAGGAA AGTCGTTGAC TTGTCAAATA ATGAAAACAT TTTTCTGCAG AACACAAATA
Ppr_3         AGAACAAATG TACCCAGGAA AGTCGTTGAC TTGTCAAATA ATGAAAACAT TTTTCTGCAG AACACAAATA
Ppr_4         AGAACAAATG TACCCAGGAA AGTCGTTGAC TTGTCAAATA ATGAAAACAT TTTTCTGCAG AACACAAATA
Ppr_5         AGAACAAATG TACCCAGGAA AGTCGTTGAC TTGTCAAATA ATGAAAACAT TTTTCTGCAG AACACAAATA

              ....|....| .
                      850
Peu_1         TCCATGAACA A
Peu_2         TCCATGAACA A
Peu_3         TCCATGAACA A
Peu_4         TCCATGAACA A
Peu_5         TCCATGAACA A
Ppr_1         TCCATGAACA A
Ppr_2         TCCATGAACA A
Ppr_3         TCCATGAACA A
Ppr_4         TCCATGAACA A
Ppr_5         TCCATGAACA A
```

Unigene38057_All (with no lysine kinase)

```
               ....|....| ....|....| ....|....| ....|....| ....|....| ....|....| ....|....|
                        10         20         30         40         50         60         70
Peu_1          GTAACAAACC CGGAGCTCTC CTCCGTAACA ATGGACGAAA GCGCCTCTTA TGACCCGACT ATAAACCACA
Peu_2          GTAACAAACC CGGAGCTCTC CTCCGTAACA ATGGACGAAA GCGCCTCTTA TGACCCGACT ATAAACCACA
Peu_3          GTAACAAACC CGGAGCTCTC CTCCGTAACA ATGGACGAAA GCGCCTCTTA TGACCCGACT ATAAACCACA
Peu_4          GTAACAAACC CGGAGCTCTC CTCCGTAACA ATGGACGAAA GCGCCTCTTA TGACCCGACT ATAAACCACA
Peu_5          GTAACAAACC CGGAGCTCTC CTCCGTAACA ATGGACGAAA GCGCCTCTTA TGACCCGACT ATAAACCACA
Ppr_1          GTAACAAACC AGGAGCTCTC CTCCGTAACA ATGGACGAAA GCGCCTCTTA TGACCCGACT ATAAACCACA
Ppr_2          GTAACAAACC AGGAGCTCTC CTCCGTAACA ATGGACGAAA GCGCCTCTTA TGACCCGACT ATAAACCACA
Ppr_3          GTAACAAACC AGGAGCTCTC CTCCGTAACA ATGGACGAAA GCGCCTCTTA TGACCCGACT ATAAACCACA
Ppr_4          GTAACAAACC AGGAGCTCTC CTCCGTAACA ATGGACGAAA GCGCCTCTTA TGACCCGACT ATAAACCACA
Ppr_5          GTAACAAACC AGGAGCTCTC CTCCGTAACA ATGGACGAAA GCGCCTCTTA TGACCCGACT ATAAACCACA

               ....|....| ....|....| ....|....| ....|....| ....|....| ....|....| ....|....|
                        80         90        100        110        120        130        140
Peu_1          TCTCCATGGA AGAGCTAAAA GTCTCCACCA AGAACTTCTC CACCGATTTA ATCATCGGCG ATGGCAGTTT
Peu_2          TCTCCATGGA AGAGCTAAAA GTCTCCACCA AGAACTTCTC CACCGATTTA ATCATCGGCG ATGGCAGTTT
Peu_3          TCTCCATGGA AGAGCTAAAA GTCTCCACCA AGAACTTCTC CACCGATTTA ATCATCGGCG ATGGCAGTTT
Peu_4          TCTCCATGGA AGAGCTAAAA GTCTCCACCA AGAACTTCTC CACCGATTTA ATCATCGGCG ATGGCAGTTT
Peu_5          TCTCCATGGA AGAGCTAAAA GTCTCCACCA AGAACTTCTC CACCGATTTA ATCATCGGCG ATGGCAGTTT
Ppr_1          TCTCCATGGA AGAGCTAAAA GTCTCCACCA AGAACTTCTC CACCGATTTA ATCATCGGCG ATGGCAGTTT
Ppr_2          TCTCCATGGA AGAGCTAAAA GTCTCCACCA AGAACTTCTC CACCGATTTA ATCATCGGCG ATGGCAGTTT
Ppr_3          TCTCCATGGA AGAGCTAAAA GTCTCCACCA AGAACTTCTC CACCGATTTA ATCATCGGCG ATGGCAGTTT
Ppr_4          TCTCCATGGA AGAGCTAAAA GTCTCCACCA AGAACTTCTC CACCGATTTA ATCATCGGCG ATGGCAGTTT
Ppr_5          TCTCCATGGA AGAGCTAAAA GTCTCCACCA AGAACTTCTC CACCGATTTA ATCATCGGCG ATGGCAGTTT

               ....|....| ....|....| ....|....| ....|....| ....|....| ....|....| ....|....|
                       150        160        170        180        190        200        210
Peu_1          CGGCTTGGTT TATAAAGCTG CACTCTACAA CGGCTCCACG GTTGCTATTA AAAAGCTTGA CCCGGACGCT
Peu_2          CGGCTTGGTT TATAAAGCTG CACTCTACAA CGGCTCCACG GTTGCTATTA AAAAGCTTGA CCCGGACGCT
Peu_3          CGGCTTGGTT TATAAAGCTG CACTCTACAA CGGCTCCACG GTTGCTATTA AAAAGCTTGA CCCGGACGCT
Peu_4          CGGCTTGGTT TATAAAGCTG CACTCTACAA CGGCTCCACG GTTGCTATTA AAAAGCTTGA CCCGGACGCT
Peu_5          CGGCTTGGTT TATAAAGCTG CACTCTACAA CGGCTCCACG GTTGCTATTA AAAAGCTTGA CCCGGACGCT
Ppr_1          CGGCTTGGTT TATAAAGCTG CACTCTACAA CGGCTCCATG GTTGCTATTA AAAAGCTTGA CCCGGACGCT
Ppr_2          CGGCTTGGTT TATAAAGCTG CACTCTACAA CGGCTCCATG GTTGCTATTA AAAAGCTTGA CCCGGACGCT
Ppr_3          CGGCTTGGTT TATAAAGCTG CACTCTACAA CGGCTCCATG GTTGCTATTA AAAAGCTTGA CCCGGACGCT
Ppr_4          CGGCTTGGTT TATAAAGCTG CACTCTACAA CGGCTCCATG GTTGCTATTA AAAAGCTTGA CCCGGACGCT
Ppr_5          CGGCTTGGTT TATAAAGCTG CACTCTACAA CGGCTCCATG GTTGCTATTA AAAAGCTTGA CCCGGACGCT

               ....|....| ....|....| ....|....| ....|....| ....|....| ....|....| ....|....|
                       220        230        240        250        260        270        280
Peu_1          TTTCAAGGTT TTCGTGAGTT TCGTGCCGAG ATGGAGACTC TAGGTCAGTT ACGCCATGGA AATATTGTCA
Peu_2          TTTCAAGGTT TTCGTGAGTT TCGTGCCGAG ATGGAGACTC TAGGTCAGTT ACGCCATGGA AATATTGTCA
Peu_3          TTTCAAGGTT TTCGTGAGTT TCGTGCCGAG ATGGAGACTC TAGGTCAGTT ACGCCATGGA AATATTGTCA
Peu_4          TTTCAAGGTT TTCGTGAGTT TCGTGCCGAG ATGGAGACTC TAGGTCAGTT ACGCCATGGA AATATTGTCA
Peu_5          TTTCAAGGTT TTCGTGAGTT TCGTGCCGAG ATGGAGACTC TAGGTCAGTT ACGCCATGGA AATATTGTCA
Ppr_1          TTTCAAGGTT TTCGTGAGTT TCGTGCCGAG ATGGAGACTC TAGGTCAGTT ACGCCATGGA AATATTGTCA
Ppr_2          TTTCAAGGTT TTCGTGAGTT TCGTGCCGAG ATGGAGACTC TAGGTCAGTT ACGCCATGGA AATATTGTCA
Ppr_3          TTTCAAGGTT TTCGTGAGTT TCGTGCCGAG ATGGAGACTC TAGGTCAGTT ACGCCATGGA AATATTGTCA
Ppr_4          TTTCAAGGTT TTCGTGAGTT TCGTGCCGAG ATGGAGACTC TAGGTCAGTT ACGCCATGGA AATATTGTCA
Ppr_5          TTTCAAGGTT TTCGTGAGTT TCGTGCCGAG ATGGAGACTC TAGGTCAGTT ACGCCATGGA AATATTGTCA

               ....|....| ....|....| ....|....| ....|....| ....|....| ....|....| ....|....|
                       290        300        310        320        330        340        350
Peu_1          AGATTCTCGG GTATTGTGTT TCGGGTCGGG ACCGGGTTTT AATTCTGGAG TTCGTTGAAA GAGGGAGTCT
Peu_2          AGATTCTCGG GTATTGTGTT TCGGGTCGGG ACCGGGTTTT AATTCTGGAG TTCGTTGAAA GAGGGAGTCT
Peu_3          AGATTCTCGG GTATTGTGTT TCGGGTCGGG ACCGGGTTTT AATTCTGGAG TTCGTTGAAA GAGGGAGTCT
Peu_4          AGATTCTCGG GTATTGTGTT TCGGGTCGGG ACCGGGTTTT AATTCTGGAG TTCGTTGAAA GAGGGAGTCT
Peu_5          AGATTCTCGG GTATTGTGTT TCGGGTCGGG ACCGGGTTTT AATTCTGGAG TTCGTTGAAA GAGGGAGTCT
Ppr_1          AGATTCTCGG GTATTGTGTT TCGGGTCGGG ACCGGGTTTT AATTCTGGAG TTCGTTGAAA GAGGGAGTCT
Ppr_2          AGATTCTCGG GTATTGTGTT TCGGGTCGGG ACCGGGTTTT AATTCTGGAG TTCGTTGAAA GAGGGAGTCT
Ppr_3          AGATTCTCGG GTATTGTGTT TCGGGTCGGG ACCGGGTTTT AATTCTGGAG TTCGTTGAAA GAGGGAGTCT
Ppr_4          AGATTCTCGG GTATTGTGTT TCGGGTCGGG ACCGGGTTTT AATTCTGGAG TTCGTTGAAA GAGGGAGTCT
```

```
Ppr_5         AGATTCTCGG GTATTGTGTT TCGGGTCGGG ACCGGGTTTT AATTCTGGAG TTCGTTGAAA GAGGGAGTCT

              ....|....| ....|....| ....|....| ....|....| ....|....| ....|....| ....|....|
                    360        370        380        390        400        410        420
Peu_1         TGACCAGTGG ATTCACGACA CATCATCAAT GGATAACGAT CACTGTGATA AGTTCCCATT ACCCTGGGAG
Peu_2         TGACCAGTGG ATTCACGACA CATCATCAAT GGATAACGAT CACTGTGATA AGTTCCCATT ACCCTGGGAG
Peu_3         TGACCAGTGG ATTCACGACA CATCATCAAT GGATAACGAT CACTGTGATA AGTTCCCATT ACCCTGGGAG
Peu_4         TGACCAGTGG ATTCACGACA CATCATCAAT GGATAACGAT CACTGTGATA AGTTCCCATT ACCCTGGGAG
Peu_5         TGACCAGTGG ATTCACGACA CATCATCAAT GGATAACGAT CACTGTGATA AGTTCCCATT ACCCTGGGAG
Ppr_1         TGACCAGTGG ATTCACGACA CATCATCAAT GGATAACGAT CACTATGATA AGCTCCCATT ACCCTGGGAC
Ppr_2         TGACCAGTGG ATTCACGACA CATCATCAAT GGATAACGAT CACTATGATA AGCTCCCATT ACCCTGGGAC
Ppr_3         TGACCAGTGG ATTCACGACA CATCATCAAT GGATAACGAT CACTATGATA AGCTCCCATT ACCCTGGGAC
Ppr_4         TGACCAGTGG ATTCACGACA CATCATCAAT GGATAACGAT CACTATGATA AGCTCCCATT ACCCTGGGAC
Ppr_5         TGACCAGTGG ATTCACGACA CATCATCAAT GGATAACGAT CACTATGATA AGCTCCCATT ACCCTGGGAC

              ....|....| ....|....| ....|....| ....|....| ....|....| ....|....| ....|....|
                    430        440        450        460        470        480        490
Peu_1         ACGAGGATCA AGATTGTTAT GGGCGTGGCC AATGGGCTTG CTTATTTGCA TGGGCTTGAT ACTCCCATTA
Peu_2         ACGAGGATCA AGATTGTTAT GGGCGTGGCC AATGGGCTTG CTTATTTGCA TGGGCTTGAT ACTCCCATTA
Peu_3         ACGAGGATCA AGATTGTTAT GGGCGTGGCC AATGGGCTTG CTTATTTGCA TGGGCTTGAT ACTCCCATTA
Peu_4         ACGAGGATCA AGATTGTTAT GGGCGTGGCC AATGGGCTTG CTTATTTGCA TGGGCTTGAT ACTCCCATTA
Peu_5         ACGAGGATCA AGATTGTTAT GGGCGTGGCC AATGGGCTTG CTTATTTGCA TGGGCTTGAT ACTCCCATTA
Ppr_1         ACGAGGATCA AGATAGTTAT GGGCGTGGCC AATGGGCTTG CTTATTTGCA TGGGCTTGAT ACTCCCATTA
Ppr_2         ACGAGGATCA AGATAGTTAT GGGCGTGGCC AATGGGCTTG CTTATTTGCA TGGGCTTGAT ACTCCCATTA
Ppr_3         ACGAGGATCA AGATAGTTAT GGGCGTGGCC AATGGGCTTG CTTATTTGCA TGGGCTTGAT ACTCCCATTA
Ppr_4         ACGAGGATCA AGATAGTTAT GGGCGTGGCC AATGGGCTTG CTTATTTGCA TGGGCTTGAT ACTCCCATTA
Ppr_5         ACGAGGATCA AGATAGTTAT GGGCGTGGCC AATGGGCTTG CTTATTTGCA TGGGCTTGAT ACTCCCATTA

              ....|....| ....|....| ....|....| ....|....| ....|....| ....|....| ....|....|
                    500        510        520        530        540        550        560
Peu_1         TTCATAGGGA TATTAAGGCT AGTAACGTTT TGTTGGACTC GAGCTTTCAG GCCCATATCT CTGATTTTGG
Peu_2         TTCATAGGGA TATTAAGGCT AGTAACGTTT TGTTGGACTC GAGCTTTCAG GCCCATATCT CTGATTTTGG
Peu_3         TTCATAGGGA TATTAAGGCT AGTAACGTTT TGTTGGACTC GAGCTTTCAG GCCCATATCT CTGATTTTGG
Peu_4         TTCATAGGGA TATTAAGGCT AGTAACGTTT TGTTGGACTC GAGCTTTCAG GCCCATATCT CTGATTTTGG
Peu_5         TTCATAGGGA TATTAAGGCT AGTAACGTTT TGTTGGACTC GAGCTTTCAG GCCCATATCT CTGATTTTGG
Ppr_1         TTCATAGGGA TATTAAGGCT AGTAACGTTT TGTTGGACTC GAGCTTTCAG GCCCATATCT CTGATTTTGG
Ppr_2         TTCATAGGGA TATTAAGGCT AGTAACGTTT TGTTGGACTC GAGCTTTCAG GCCCATATCT CTGATTTTGG
Ppr_3         TTCATAGGGA TATTAAGGCT AGTAACGTTT TGTTGGACTC GAGCTTTCAG GCCCATATCT CTGATTTTGG
Ppr_4         TTCATAGGGA TATTAAGGCT AGTAACGTTT TGTTGGACTC GAGCTTTCAG GCCCATATCT CTGATTTTGG
Ppr_5         TTCATAGGGA TATTAAGGCT AGTAACGTTT TGTTGGACTC GAGCTTTCAG GCCCATATCT CTGATTTTGG

              ....|....|
                    570
Peu_1         GCTTGCCCGT
Peu_2         GCTTGCCCGT
Peu_3         GCTTGCCCGT
Peu_4         GCTTGCCCGT
Peu_5         GCTTGCCCGT
Ppr_1         GCTTGCCCGT
Ppr_2         GCTTGCCCGT
Ppr_3         GCTTGCCCGT
Ppr_4         GCTTGCCCGT
Ppr_5         GCTTGCCCGT
```

Unigene7669_All (ATSIK; ATP binding / kinase/ protein kinase/ protein tyrosine kinase)

```
              ....|....| ....|....| ....|....| ....|....| ....|....| ....|....| ....|....|
                      10         20         30         40         50         60         70
Peu_1         AAACAGGTGG GAATTGTAGG GTTATATGGA ATGGGGGGTG TCGGAAAAAC TACCCTCTTG ACTCAAATCA
Peu_2         AAACAGGTGG GAATTGTAGG GTTATATGGA ATGGGGGGTG TCGGAAAAAC TACCCTCTTG ACTCAAATCA
Peu_3         AAACAGGTGG GAATTGTAGG GTTATATGGA ATGGGGGGTG TCGGAAAAAC TACCCTCTTG ACTCAAATCA
Peu_4         AAACAGGTGG GAATTGTAGG GTTATATGGA ATGGGGGGTG TCGGAAAAAC TACCCTCTTG ACTCAAATCA
Peu_5         AAACAGGTGG GAATTGTAGG GTTATATGGA ATGGGGGGTG TCGGAAAAAC TACCCTCTTG ACTCAAATCA
Ppr_1         AAACAGGTGG GAATTGTAGG GTTATATGGA ATGGGGGGTG TCGGAAAAAC TACCCTCTTG ACTCAAATCA
Ppr_2         AAACAGGTGG GAATTGTAGG GTTATATGGA ATGGGGGGTG TCGGAAAAAC TACCCTCTTG ACTCAAATCA

              ....|....| ....|....| ....|....| ....|....| ....|....| ....|....| ....|....|
                      80         90        100        110        120        130        140
Peu_1         ACAACGAGTC TCTCAAGACA CTCGATTATT TTGATATTGT GATATGGGTA GTGGTGTCAA AAGATTTGAA
Peu_2         ACAACGAGTC TCTCAAGACA CTCGATTATT TTGATATTGT GATATGGGTA GTGGTGTCAA AAGATTTGAA
Peu_3         ACAACGAGTC TCTCAAGACA CTCGATTATT TTGATATTGT GATATGGGTA GTGGTGTCAA AAGATTTGAA
Peu_4         ACAACGAGTC TCTCAAGACA CTCGATTATT TTGATATTGT GATATGGGTA GTGGTGTCAA AAGATTTGAA
Peu_5         ACAACGAGTC TCTCAAGACA CTCGATTATT TTGATATTGT GATATGGGTA GTGGTGTCAA AAGATTTGAA
Ppr_1         ACAACGAGTC TCTCAAGACA CTCGATTATT TTGATATTGT GATATGGGTA GTGGTGTCAA AAGATTTGAA
Ppr_2         ACAACGAGTC TCTCAAGACA CTCGATTATT TTGATATTGT GATATGGGTA GTGGTGTCAA AAGATTTGAA

              ....|....| ....|....| ....|....| ....|....| ....|....| ....|....| ....|....|
                     150        160        170        180        190        200        210
Peu_1         ACTTAACACT ATTCAAGAAA GCATAGGGAG AAAAATAGGC TGTTCTGATG ATTTGTGGAA GAATAAAAGC
Peu_2         ACTTAACACT ATTCAAGAAA GCATAGGGAG AAAAATAGGC TGTTCTGATG ATTTGTGGAA GAATAAAAGC
Peu_3         ACTTAACACT ATTCAAGAAA GCATAGGGAG AAAAATAGGC TGTTCTGATG ATTTGTGGAA GAATAAAAGC
Peu_4         ACTTAACACT ATTCAAGAAA GCATAGGGAG AAAAATAGGC TGTTCTGATG ATTTGTGGAA GAATAAAAGC
Peu_5         ACTTAACACT ATTCAAGAAA GCATAGGGAG AAAAATAGGC TGTTCTGATG ATTTGTGGAA GAATAAAAGC
Ppr_1         ACTTAACACT ATTCAAGAAA GCATAGGGAG AAAAATAGGC TGTTCTGATG ATTTGTGGAA GAATAAAAGC
Ppr_2         ACTTAACACT ATTCAAGAAA GCATAGGGAG AAAAATAGGC TGTTCTGATG ATTTGTGGAA GAATAAAAGC

              ....|....| ....|....| ....|....| ....|....| ....|....| ....|....| ....|....|
                     220        230        240        250        260        270        280
Peu_1         CTTGACGAAA AATCTGTGGA CATATTTACT GCCTTGCGCC ATAAAAGGTT TGTGATGTTG TTGGATGATA
Peu_2         CTTGACGAAA AATCTGTGGA CATATTTACT GCCTTGCGCC ATAAAAGGTT TGTGATGTTG TTGGATGATA
Peu_3         CTTGACGAAA AATCTGTGGA CATATTTACT GCCTTGCGCC ATAAAAGGTT TGTGATGTTG TTGGATGATA
Peu_4         CTTGACGAAA AATCTGTGGA CATATTTACT GCCTTGCGCC ATAAAAGGTT TGTGATGTTG TTGGATGATA
Peu_5         CTTGACGAAA AATCTGTGGA CATATTTACT GCCTTGCGCC ATAAAAGGTT TGTGATGTTG TTGGATGATA
Ppr_1         CTTGACGAAA AAGCTGTGGA CATATTTACT GCCTTACGCC ATAAAAGGTT TGTGATGTTG TTGGATGATA
Ppr_2         CTTGACGAAA AAGCTGTGGA CATATTTACT GCCTTACGCC ATAAAAGGTT TGTGATGTTG TTGGATGATA

              ....|....| ....|....| ....|....| ....|....| ....|....| ....|....| ....|....|
                     290        300        310        320        330        340        350
Peu_1         TCTGGGAGAG GGTTGATTTA AAAAAACTTG GAGTTCCTCT TCCTGATATG AAAAACGGTT CCAAGGTAGT
Peu_2         TCTGGGAGAG GGTTGATTTA AAAAAACTTG GAGTTCCTCT TCCTGATATG AAAAACGGTT CCAAGGTAGT
Peu_3         TCTGGGAGAG GGTTGATTTA AAAAAACTTG GAGTTCCTCT TCCTGATATG AAAAACGGTT CCAAGGTAGT
Peu_4         TCTGGGAGAG GGTTGATTTA AAAAAACTTG GAGTTCCTCT TCCTGATATG AAAAACGGTT CCAAGGTAGT
Peu_5         TCTGGGAGAG GGTTGATTTA AAAAAACTTG GAGTTCCTCT TCCTGATATG AAAAACGGTT CCAAGGTAGT
Ppr_1         TCTGGGAGAG GGTTGATTTA AAAAAACTTG GAGTTCCTCT TCCTGATATG AAAAACGGTT CCAAGGTAGT
Ppr_2         TCTGGGAGAG GGTTGATTTA AAAAAACTTG GAGTTCCTCT TCCTGATATG AAAAACGGTT CCAAGGTAGT

              ....|...

Peu_1         TTTCACAA
Peu_2         TTTCACAA
Peu_3         TTTCACAA
Peu_4         TTTCACAA
Peu_5         TTTCACAA
Ppr_1         TTTCACAA
Ppr_2         TTTCACAA
```

Unigene25055_All (twin LOV protein 1)

```
                ....|....| ....|....| ....|....| ....|....| ....|....| ....|....| ....|....|
                         10         20         30         40         50         60         70
Peu_1           TCTCTCCATC TCCAGTTTTT TATGCTAGAA ATAAATTAAG AGGAAATTGA GTTGCCATTT TAGGCCATCT
Peu_2           TCTCTCCATC TCCAGTTTTT TATGCTAGAA ATAAATTAAG AGGAAATTGA GTTGCCATTT TAGGCCATCT
Peu_3           TCTCTCCATC TCCAGTTTTT TATGCTAGAA ATAAATTAAG AGGAAATTGA GTTGCCATTT TAGGCCATCT
Peu_4           TCTCTCCATC TCCAGTTTTT TATGCTAGAA ATAAATTAAG AGGAAATTGA GTTGCCATTT TAGGCCATCT
Peu_5           TCTCTCCATC TCCAGTTTTT TATGCTAGAA ATAAATTAAG AGGAAATTGA GTTGCCATTT TAGGCCATCT
Ppr_1           TCTCTCCATC TCCAGTTTTT TATGCTAGAA ATAAATTAAG AGGAAATTGG GTTGCCATTT TAGGCCATCT
Ppr_2           TCTCTCCATC TCCAGTTTTT TATGCTAGAA ATAAATTAAG AGGAAATTGG GTTGCCATTT TAGGCCATCT
Ppr_3           TCTCTCCATC TCCAGTTTTT TATGCTAGAA ATAAATTAAG AGGAAATTGG GTTGCCATTT TAGGCCATCT
Ppr_4           TCTCTCCATC TCCAGTTTTT TATGCTAGAA ATAAATTAAG AGGAAATTGG GTTGCCATTT TAGGCCATCT
Ppr_5           TCTCTCCATC TCCAGTTTTT TATGCTAGAA ATAAATTAAG AGGAAATTGG GTTGCCATTT TAGGCCATCT

                ....|....| ....|....| ....|....| ....|....| ....|....| ....|....| ....|....|
                         80         90        100        110        120        130        140
Peu_1           GGATACGGAA TCCAGACCCT TTTATTGTAT TTTATTTCTT ACAATATGTG CGATAATTAT ACTGTATTTA
Peu_2           GGATACGGAA TCCAGACCCT TTTATTGTAT TTTATTTCTT ACAATATGTG CGATAATTAT ACTGTATTTA
Peu_3           GGATACGGAA TCCAGACCCT TTTATTGTAT TTTATTTCTT ACAATATGTG CGATAATTAT ACTGTATTTA
Peu_4           GGATACGGAA TCCAGACCCT TTTATTGTAT TTTATTTCTT ACAATATGTG CGATAATTAT ACTGTATTTA
Peu_5           GGATACGGAA TCCAGACCCT TTTATTGTAT TTTATTTCTT ACAATATGTG CGATAATTAT ACTGTATTTA
Ppr_1           GGATACGGAA TCCAGACCCT TTTATTGTAT TTTATTTCTT ACAATATGTG CGATAATTAT ACTGTATTTA
Ppr_2           GGATACGGAA TCCAGACCCT TTTATTGTAT TTTATTTCTT ACAATATGTG CGATAATTAT ACTGTATTTA
Ppr_3           GGATACGGAA TCCAGACCCT TTTATTGTAT TTTATTTCTT ACAATATGTG CGATAATTAT ACTGTATTTA
Ppr_4           GGATACGGAA TCCAGACCCT TTTATTGTAT TTTATTTCTT ACAATATGTG CGATAATTAT ACTGTATTTA
Ppr_5           GGATACGGAA TCCAGACCCT TTTATTGTAT TTTATTTCTT ACAATATGTG CGATAATTAT ACTGTATTTA

                ....|....| ....|....| ....|....| ....|....| ....|....| ....|....| ....|....|
                        150        160        170        180        190        200        210
Peu_1           CATTATTAAT GATATTTGCT TTTGGGCATG ACAGAATCGA AGAAAGTACG TGTAGATTTA CCTCCTGAAC
Peu_2           CATTATTAAT GATATTTGCT TTTGGGCATG ACAGAATCGA AGAAAGTACG TGTAGATTTA CCTCCTGAAC
Peu_3           CATTATTAAT GATATTTGCT TTTGGGCATG ACAGAATCGA AGAAAGTACG TGTAGATTTA CCTCCTGAAC
Peu_4           CATTATTAAT GATATTTGCT TTTGGGCATG ACAGAATCGA AGAAAGTACG TGTAGATTTA CCTCCTGAAC
Peu_5           CATTATTAAT GATATTTGCT TTTGGGCATG ACAGAATCGA AGAAAGTACG TGTAGATTTA CCTCCTGAAC
Ppr_1           CATTATTAAT GATATTTGCT TTTGGGCGTG ACAGAATCGA AGAAAGTACG TGTAGATTTG CCTCCTGAAC
Ppr_2           CATTATTAAT GATATTTGCT TTTGGGCGTG ACAGAATCGA AGAAAGTACG TGTAGATTTG CCTCCTGAAC
Ppr_3           CATTATTAAT GATATTTGCT TTTGGGCGTG ACAGAATCGA AGAAAGTACG TGTAGATTTG CCTCCTGAAC
Ppr_4           CATTATTAAT GATATTTGCT TTTGGGCGTG ACAGAATCGA AGAAAGTACG TGTAGATTTG CCTCCTGAAC
Ppr_5           CATTATTAAT GATATTTGCT TTTGGGCGTG ACAGAATCGA AGAAAGTACG TGTAGATTTG CCTCCTGAAC

                ....|....| ....|....| ....|....| ....|....| ....|....| ....|....| ....|....|
                        220        230        240        250        260        270        280
Peu_1           TTTGTGACGT AATTTTATCT CCAGTATCAA TCAGCACTCT TTATTCTTAT ACATTTATCC CATCAATAAT
Peu_2           TTTGTGACGT AATTTTATCT CCAGTATCAA TCAGCACTCT TTATTCTTAT ACATTTATCC CATCAATAAT
Peu_3           TTTGTGACGT AATTTTATCT CCAGTATCAA TCAGCACTCT TTATTCTTAT ACATTTATCC CATCAATAAT
Peu_4           TTTGTGACGT AATTTTATCT CCAGTATCAA TCAGCACTCT TTATTCTTAT ACATTTATCC CATCAATAAT
Peu_5           TTTGTGACGT AATTTTATCT CCAGTATCAA TCAGCACTCT TTATTCTTAT ACATTTATCC CATCAATAAT
Ppr_1           TTTGTGAYGT AATTTTATCT CCAGTATCAA TCAGCACTCT TTATTCTTAT ACATTTATCC CATCAATAAT
Ppr_2           TTTGTGAYGT AATTTTATCT CCAGTATCAA TCAGCACTCT TTATTCTTAT ACATTTATCC CATCAATAAT
Ppr_3           TTTGTGAYGT AATTTTATCT CCAGTATCAA TCAGCACTCT TTATTCTTAT ACATTTATCC CATCAATAAT
Ppr_4           TTTGTGAYGT AATTTTATCT CCAGTATCAA TCAGCACTCT TTATTCTTAT ACATTTATCC CATCAATAAT
Ppr_5           TTTGTGAYGT AATTTTATCT CCAGTATCAA TCAGCACTCT TTATTCTTAT ACATTTATCC CATCAATAAT

                ....|....| ....|....| ....|....| ....|....| ....|....| ....|....| ....|....|
                        290        300        310        320        330        340        350
Peu_1           GCATCGACTA GAGTCCCTGC TTATTGCTGT CAACTTAAAG AAGATGCATT CGGATCATTG CATGCAAAAT
Peu_2           GCATCGACTA GAGTCCCTGC TTATTGCTGT CAACTTAAAG AAGATGCATT CGGATCATTG CATGCAAAAT
Peu_3           GCATCGACTA GAGTCCCTGC TTATTGCTGT CAACTTAAAG AAGATGCATT CGGATCATTG CATGCAAAAT
Peu_4           GCATCGACTA GAGTCCCTGC TTATTGCTGT CAACTTAAAG AAGATGCATT CGGATCATTG CATGCAAAAT
Peu_5           GCATCGACTA GAGTCCCTGC TTATTGCTGT CAACTTAAAG AAGATGCATT CGGATCATTG CATGCAAAAT
Ppr_1           GCATCGACTA GAGTCCCTGC TTATTGCTGT CAACTTAAAG AAGATGCATT CGGATCATTG CATGCAAAAT
Ppr_2           GCATCGACTA GAGTCCCTGC TTATTGCTGT CAACTTAAAG AAGATGCATT CGGATCATTG CATGCAAAAT
Ppr_3           GCATCGACTA GAGTCCCTGC TTATTGCTGT CAACTTAAAG AAGATGCATT CGGATCATTG CATGCAAAAT
Ppr_4           GCATCGACTA GAGTCCCTGC TTATTGCTGT CAACTTAAAG AAGATGCATT CGGATCATTG CATGCAAAAT
```

```
Ppr_5          GCATCGACTA GAGTCCCTGC TTATTGCTGT CAACTTAAAG AAGATGCATT CGGATCATTG CATGCAAAAT


               ....|....| ....|....| ....|....| ....|....| ....|....| ....|....| ....|....|
                       360        370        380        390        400        410        420
Peu_1          GTTGATATTC CAGCCATGAA GGTAATTTAT TCCGACTCTA ATTATGCCAA ATGTTTTGTT TTTTGGACTT
Peu_2          GTTGATATTC CAGCCATGAA GGTAATTTAT TCCGACTCTA ATTATGCCAA ATGTTTTGTT TTTTGGACTT
Peu_3          GTTGATATTC CAGCCATGAA GGTAATTTAT TCCGACTCTA ATTATGCCAA ATGTTTTGTT TTTTGGACTT
Peu_4          GTTGATATTC CAGCCATGAA GGTAATTTAT TCCGACTCTA ATTATGCCAA ATGTTTTGTT TTTTGGACTT
Peu_5          GTTGATATTC CAGCCATGAA GGTAATTTAT TCCGACTCTA ATTATGCCAA ATGTTTTGTT TTTTGGACTT
Ppr_1          GTTGATATTC CAGCCATGAA GGTAATTTAT TCTGACTCTA ATTTTGCCAA ATGTTTTGTT TTTTGGACTT
Ppr_2          GTTGATATTC CAGCCATGAA GGTAATTTAT TCTGACTCTA ATTTTGCCAA ATGTTTTGTT TTTTGGACTT
Ppr_3          GTTGATATTC CAGCCATGAA GGTAATTTAT TCTGACTCTA ATTTTGCCAA ATGTTTTGTT TTTTGGACTT
Ppr_4          GTTGATATTC CAGCCATGAA GGTAATTTAT TCTGACTCTA ATTTTGCCAA ATGTTTTGTT TTTTGGACTT
Ppr_5          GTTGATATTC CAGCCATGAA GGTAATTTAT TCTGACTCTA ATTTTGCCAA ATGTTTTGTT TTTTGGACTT


               ....|....| ....|....| ....|....| ....|....| ....|....| ....|....| ....|....|
                       430        440        450        460        470        480        490
Peu_1          GTACTGACCA AACTTTATTA TATGGTAGGT ATTGGAAGCA ATTACCACAA AGAAGTGCCA GGAGAAATTT
Peu_2          GTACTGACCA AACTTTATTA TATGGTAGGT ATTGGAAGCA ATTACCACAA AGAAGTGCCA GGAGAAATTT
Peu_3          GTACTGACCA AACTTTATTA TATGGTAGGT ATTGGAAGCA ATTACCACAA AGAAGTGCCA GGAGAAATTT
Peu_4          GTACTGACCA AACTTTATTA TATGGTAGGT ATTGGAAGCA ATTACCACAA AGAAGTGCCA GGAGAAATTT
Peu_5          GTACTGACCA AACTTTATTA TATGGTAGGT ATTGGAAGCA ATTACCACAA AGAAGTGCCA GGAGAAATTT
Ppr_1          GTACTGACCA AACTTTATTA TATGGTAGGT ATTGGAAGCA ATTACCACAA AGAAGTGCCA GGAGAAATTT
Ppr_2          GTACTGACCA AACTTTATTA TATGGTAGGT ATTGGAAGCA ATTACCACAA AGAAGTGCCA GGAGAAATTT
Ppr_3          GTACTGACCA AACTTTATTA TATGGTAGGT ATTGGAAGCA ATTACCACAA AGAAGTGCCA GGAGAAATTT
Ppr_4          GTACTGACCA AACTTTATTA TATGGTAGGT ATTGGAAGCA ATTACCACAA AGAAGTGCCA GGAGAAATTT
Ppr_5          GTACTGACCA AACTTTATTA TATGGTAGGT ATTGGAAGCA ATTACCACAA AGAAGTGCCA GGAGAAATTT


               ....|....| ....|....| ..
                       500        510
Peu_1          CATTTGGAAT CCTTAGAAAC TC
Peu_2          CATTTGGAAT CCTTAGAAAC TC
Peu_3          CATTTGGAAT CCTTAGAAAC TC
Peu_4          CATTTGGAAT CCTTAGAAAC TC
Peu_5          CATTTGGAAT CCTTAGAAAC TC
Ppr_1          CATTTGGAAT CCTTAGAAAC TC
Ppr_2          CATTTGGAAT CCTTAGAAAC TC
Ppr_3          CATTTGGAAT CCTTAGAAAC TC
Ppr_4          CATTTGGAAT CCTTAGAAAC TC
Ppr_5          CATTTGGAAT CCTTAGAAAC TC
```

Unigene30965_All (NA)

```
                ....|....| ....|....| ....|....| ....|....| ....|....| ....|....| ....|....|
                         10         20         30         40         50         60         70
Peu_1           TCCAGAAGTC CCGCTCTTTC TATCCACTCG TTATCTTCCT CCAGCTCCCC TCCTRCCTCC AAAGAAGAAG
Peu_2           TCCAGAAGTC CCGCTCTTTC TATCCACTCG TTATCTTCCT CCAGCTCCCC TCCTRCCTCC AAAGAAGAAG
Peu_3           TCCAGAAGTC CCGCTCTTTC TATCCACTCG TTATCTTCCT CCAGCTCCCC TCCTRCCTCC AAAGAAGAAG
Peu_4           TCCAGAAGTC CCGCTCTTTC TATCCACTCG TTATCTTCCT CCAGCTCCCC TCCTRCCTCC AAAGAAGAAG
Peu_5           TCCAGAAGTC CCGCTCTTTC TATCCACTCG TTATCTTCCT CCAGCTCCCC TCCTRCCTCC AAAGAAGAAG
Ppr_1           TCCAGAAGTC CCGCTCTTTC TATCCACTCS TTATCTTCCT CCAGCTCCCC TCCTACCTCC AAAGAAGAAG
Ppr_2           TCCAGAAGTC CCGCTCTTTC TATCCACTCS TTATCTTCCT CCAGCTCCCC TCCTACCTCC AAAGAAGAAG
Ppr_3           TCCAGAAGTC CCGCTCTTTC TATCCACTCS TTATCTTCCT CCAGCTCCCC TCCTACCTCC AAAGAAGAAG
Ppr_4           TCCAGAAGTC CCGCTCTTTC TATCCACTCS TTATCTTCCT CCAGCTCCCC TCCTACCTCC AAAGAAGAAG
Ppr_5           TCCAGAAGTC CCGCTCTTTC TATCCACTCS TTATCTTCCT CCAGCTCCCC TCCTACCTCC AAAGAAGAAG

                ....|....| ....|....| ....|....| ....|....| ....|....| ....|....| ....|....|
                         80         90        100        110        120        130        140
Peu_1           CCATTGTTCA AGCCAAAACC TGTCTCTCAA CCACCCTAGA GAAGCCCCTA AACAACCCAA AACTAGCTGG
Peu_2           CCATTGTTCA AGCCAAAACC TGTCTCTCAA CCACCCTAGA GAAGCCCCTA AACAACCCAA AACTAGCTGG
Peu_3           CCATTGTTCA AGCCAAAACC TGTCTCTCAA CCACCCTAGA GAAGCCCCTA AACAACCCAA AACTAGCTGG
Peu_4           CCATTGTTCA AGCCAAAACC TGTCTCTCAA CCACCCTAGA GAAGCCCCTA AACAACCCAA AACTAGCTGG
Peu_5           CCATTGTTCA AGCCAAAACC TGTCTCTCAA CCACCCTAGA GAAGCCCCTA AACAACCCAA AACTAGCTGG
Ppr_1           CCATTGTTCA AGCCAAAACC TGTCTCTCAA CCACCCTAGA GAAGCCCCTA AACAACCCAA AAYTAGCTGG
Ppr_2           CCATTGTTCA AGCCAAAACC TGTCTCTCAA CCACCCTAGA GAAGCCCCTA AACAACCCAA AAYTAGCTGG
Ppr_3           CCATTGTTCA AGCCAAAACC TGTCTCTCAA CCACCCTAGA GAAGCCCCTA AACAACCCAA AAYTAGCTGG
Ppr_4           CCATTGTTCA AGCCAAAACC TGTCTCTCAA CCACCCTAGA GAAGCCCCTA AACAACCCAA AAYTAGCTGG
Ppr_5           CCATTGTTCA AGCCAAAACC TGTCTCTCAA CCACCCTAGA GAAGCCCCTA AACAACCCAA AAYTAGCTGG

                ....|....| ....|....| ....|....| ....|....| ....|....| ....|....| ....|....|
                        150        160        170        180        190        200        210
Peu_1           CAAACTCAAG AAACTGAAGC AACCTAGATT CCAGGTAGAA ATCCCAGTCA TAGATGACTC GCCAACTTCT
Peu_2           CAAACTCAAG AAACTGAAGC AACCTAGATT CCAGGTAGAA ATCCCAGTCA TAGATGACTC GCCAACTTCT
Peu_3           CAAACTCAAG AAACTGAAGC AACCTAGATT CCAGGTAGAA ATCCCAGTCA TAGATGACTC GCCAACTTCT
Peu_4           CAAACTCAAG AAACTGAAGC AACCTAGATT CCAGGTAGAA ATCCCAGTCA TAGATGACTC GCCAACTTCT
Peu_5           CAAACTCAAG AAACTGAAGC AACCTAGATT CCAGGTAGAA ATCCCAGTCA TAGATGACTC GCCAACTTCT
Ppr_1           CAAACTCAAG AAACTGAAGC AACCTAGATT CCAGGTAGAA ATCCCAGTCA TRGATGACTC GCCAACTTCT
Ppr_2           CAAACTCAAG AAACTGAAGC AACCTAGATT CCAGGTAGAA ATCCCAGTCA TRGATGACTC GCCAACTTCT
Ppr_3           CAAACTCAAG AAACTGAAGC AACCTAGATT CCAGGTAGAA ATCCCAGTCA TRGATGACTC GCCAACTTCT
Ppr_4           CAAACTCAAG AAACTGAAGC AACCTAGATT CCAGGTAGAA ATCCCAGTCA TRGATGACTC GCCAACTTCT
Ppr_5           CAAACTCAAG AAACTGAAGC AACCTAGATT CCAGGTAGAA ATCCCAGTCA TRGATGACTC GCCAACTTCT

                ....|....| ....|....| ....|....| ....|....| ....|....| ....|....| ....|....|
                        220        230        240        250        260        270        280
Peu_1           CTCTCTCAAC TTGCCTCTGA TGTTTTCAAA GACCTGCCCA TTAAGAGAAG AGGGACACCG GTCAAGATTT
Peu_2           CTCTCTCAAC TTGCCTCTGA TGTTTTCAAA GACCTGCCCA TTAAGAGAAG AGGGACACCG GTCAAGATTT
Peu_3           CTCTCTCAAC TTGCCTCTGA TGTTTTCAAA GACCTGCCCA TTAAGAGAAG AGGGACACCG GTCAAGATTT
Peu_4           CTCTCTCAAC TTGCCTCTGA TGTTTTCAAA GACCTGCCCA TTAAGAGAAG AGGGACACCG GTCAAGATTT
Peu_5           CTCTCTCAAC TTGCCTCTGA TGTTTTCAAA GACCTGCCCA TTAAGAGAAG AGGGACACCG GTCAAGATTT
Ppr_1           CTCTCTCAAC TTGCCTCTGA TGTTTTCAAA GACCTGCCCA TTAAGAGAAG AGGGACACCG GTCAAGATTT
Ppr_2           CTCTCTCAAC TTGCCTCTGA TGTTTTCAAA GACCTGCCCA TTAAGAGAAG AGGGACACCG GTCAAGATTT
Ppr_3           CTCTCTCAAC TTGCCTCTGA TGTTTTCAAA GACCTGCCCA TTAAGAGAAG AGGGACACCG GTCAAGATTT
Ppr_4           CTCTCTCAAC TTGCCTCTGA TGTTTTCAAA GACCTGCCCA TTAAGAGAAG AGGGACACCG GTCAAGATTT
Ppr_5           CTCTCTCAAC TTGCCTCTGA TGTTTTCAAA GACCTGCCCA TTAAGAGAAG AGGGACACCG GTCAAGATTT

                ....|....| ....|....| ....|....| ....|....| ....|....| ....|....| ....|....|
                        290        300        310        320        330        340        350
Peu_1           TAATCCTCTG GCCGAGTCCC ACATTGAAGA ACAGAGGCAT AGAAGCATTT CAATCTTACA ATTCATCAAA
Peu_2           TAATCCTCTG GCCGAGTCCC ACATTGAAGA ACAGAGGCAT AGAAGCATTT CAATCTTACA ATTCATCAAA
Peu_3           TAATCCTCTG GCCGAGTCCC ACATTGAAGA ACAGAGGCAT AGAAGCATTT CAATCTTACA ATTCATCAAA
Peu_4           TAATCCTCTG GCCGAGTCCC ACATTGAAGA ACAGAGGCAT AGAAGCATTT CAATCTTACA ATTCATCAAA
Peu_5           TAATCCTCTG GCCGAGTCCC ACATTGAAGA ACAGAGGCAT AGAAGCATTT CAATCTTACA ATTCATCAAA
Ppr_1           TAATCCTCTG GCCGAGTCCC ACATTRAAGA ACAGAGGCAT AGAAGCATTT CAATCTTACA ATTCATCAAA
Ppr_2           TAATCCTCTG GCCGAGTCCC ACATTRAAGA ACAGAGGCAT AGAAGCATTT CAATCTTACA ATTCATCAAA
Ppr_3           TAATCCTCTG GCCGAGTCCC ACATTRAAGA ACAGAGGCAT AGAAGCATTT CAATCTTACA ATTCATCAAA
Ppr_4           TAATCCTCTG GCCGAGTCCC ACATTRAAGA ACAGAGGCAT AGAAGCATTT CAATCTTACA ATTCATCAAA
Ppr_5           TAATCCTCTG GCCGAGTCCC ACATTRAAGA ACAGAGGCAT AGAAGCATTT CAATCTTACA ATTCATCAAA

                ....|....| ....|....| ....|....| ....|....| ....|....| ....|....| ....|....|
                        360        370        380        390        400        410        420
```

```
Peu_1          AAATGTTGAG CACGTCGACG TTTCTTCAGT CAGAAATACA GACAACAGAA TTTTGAGTTC TGCCGAAGTG
Peu_2          AAATGTTGAG CACGTCGACG TTTCTTCAGT CAGAAATACA GACAACAGAA TTTTGAGTTC TGCCGAAGTG
Peu_3          AAATGTTGAG CACGTCGACG TTTCTTCAGT CAGAAATACA GACAACAGAA TTTTGAGTTC TGCCGAAGTG
Peu_4          AAATGTTGAG CACGTCGACG TTTCTTCAGT CAGAAATACA GACAACAGAA TTTTGAGTTC TGCCGAAGTG
Peu_5          AAATGTTGAG CACGTCGACG TTTCTTCAGT CAGAAATACA GACAACAGAA TTTTGAGTTC TGCCGAAGTG
Ppr_1          AAATGTTGAG CACGTCGACG TTTCTTCAGT CAGAAATACA GACAACAGAA TTTTGAGTTC TGCCGAAGTG
Ppr_2          AAATGTTGAG CACGTCGACG TTTCTTCAGT CAGAAATACA GACAACAGAA TTTTGAGTTC TGCCGAAGTG
Ppr_3          AAATGTTGAG CACGTCGACG TTTCTTCAGT CAGAAATACA GACAACAGAA TTTTGAGTTC TGCCGAAGTG
Ppr_4          AAATGTTGAG CACGTCGACG TTTCTTCAGT CAGAAATACA GACAACAGAA TTTTGAGTTC TGCCGAAGTG
Ppr_5          AAATGTTGAG CACGTCGACG TTTCTTCAGT CAGAAATACA GACAACAGAA TTTTGAGTTC TGCCGAAGTG

               ....|....| ....|....| ....|....| ....|....| ....|....| ....|....| ....|....|
                       430        440        450        460        470        480        490
Peu_1          GCAGTGTTTC TGGGGCCAGA GACTTCACAG TTGCCTGATA TCAAAACTGT TACTGATATT CTGTACCCAA
Peu_2          GCAGTGTTTC TGGGGCCAGA GACTTCACAG TTGCCTGATA TCAAAACTGT TACTGATATT CTGTACCCAA
Peu_3          GCAGTGTTTC TGGGGCCAGA GACTTCACAG TTGCCTGATA TCAAAACTGT TACTGATATT CTGTACCCAA
Peu_4          GCAGTGTTTC TGGGGCCAGA GACTTCACAG TTGCCTGATA TCAAAACTGT TACTGATATT CTGTACCCAA
Peu_5          GCAGTGTTTC TGGGGCCAGA GACTTCACAG TTGCCTGATA TCAAAACTGT TACTGATATT CTGTACCCAA
Ppr_1          GCAGTGTTTC TGGGGCCAGA GACTTCACAG TTGCCTGATA TCAAAACTGT TACTGATATT TTGTACCCAA
Ppr_2          GCAGTGTTTC TGGGGCCAGA GACTTCACAG TTGCCTGATA TCAAAACTGT TACTGATATT TTGTACCCAA
Ppr_3          GCAGTGTTTC TGGGGCCAGA GACTTCACAG TTGCCTGATA TCAAAACTGT TACTGATATT TTGTACCCAA
Ppr_4          GCAGTGTTTC TGGGGCCAGA GACTTCACAG TTGCCTGATA TCAAAACTGT TACTGATATT TTGTACCCAA
Ppr_5          GCAGTGTTTC TGGGGCCAGA GACTTCACAG TTGCCTGATA TCAAAACTGT TACTGATATT TTGTACCCAA

               ....|....| ....|....| ....|....| ....|....| ....|....| ....|....| ....|....|
                       500        510        520        530        540        550        560
Peu_1          AGCCAGTAGT CATATTCAAC CCCAAATGGG GGTTTGGAGA GGAGAACGAG TTTGGTGATT TGAGTGGCTT
Peu_2          AGCCAGTAGT CATATTCAAC CCCAAATGGG GGTTTGGAGA GGAGAACGAG TTTGGTGATT TGAGTGGCTT
Peu_3          AGCCAGTAGT CATATTCAAC CCCAAATGGG GGTTTGGAGA GGAGAACGAG TTTGGTGATT TGAGTGGCTT
Peu_4          AGCCAGTAGT CATATTCAAC CCCAAATGGG GGTTTGGAGA GGAGAACGAG TTTGGTGATT TGAGTGGCTT
Peu_5          AGCCAGTAGT CATATTCAAC CCCAAATGGG GGTTTGGAGA GGAGAACGAG TTTGGTGATT TGAGTGGCTT
Ppr_1          AGCCAGTAGT CATATTCAAC CCCAAATGGG GGTTTGGAGA GGAGAACGAG TTTGGTGATT TGAGTGGYTT
Ppr_2          AGCCAGTAGT CATATTCAAC CCCAAATGGG GGTTTGGAGA GGAGAACGAG TTTGGTGATT TGAGTGGYTT
Ppr_3          AGCCAGTAGT CATATTCAAC CCCAAATGGG GGTTTGGAGA GGAGAACGAG TTTGGTGATT TGAGTGGYTT
Ppr_4          AGCCAGTAGT CATATTCAAC CCCAAATGGG GGTTTGGAGA GGAGAACGAG TTTGGTGATT TGAGTGGYTT
Ppr_5          AGCCAGTAGT CATATTCAAC CCCAAATGGG GGTTTGGAGA GGAGAACGAG TTTGGTGATT TGAGTGGYTT

               ....|....| ....|....| ....|....| ....|....| ....|....| ....|....| ....|....|
                       570        580        590        600        610        620        630
Peu_1          TGTGGGATCC TTTGAAGTGG TATATTCATT CATGGGGTTG GAGGTTAGAG GTATTTTGAG CAAGCGAAAA
Peu_2          TGTGGGATCC TTTGAAGTGG TATATTCATT CATGGGGTTG GAGGTTAGAG GTATTTTGAG CAAGCGAAAA
Peu_3          TGTGGGATCC TTTGAAGTGG TATATTCATT CATGGGGTTG GAGGTTAGAG GTATTTTGAG CAAGCGAAAA
Peu_4          TGTGGGATCC TTTGAAGTGG TATATTCATT CATGGGGTTG GAGGTTAGAG GTATTTTGAG CAAGCGAAAA
Peu_5          TGTGGGATCC TTTGAAGTGG TATATTCATT CATGGGGTTG GAGGTTAGAG GTATTTTGAG CAAGCGAAAA
Ppr_1          TGTGGGATCC TTTGAAGTGG TATATTCATT CATGGGGTTG GAGGTTAGAG GTATTTTGAG CAAGCGAAAA
Ppr_2          TGTGGGATCC TTTGAAGTGG TATATTCATT CATGGGGTTG GAGGTTAGAG GTATTTTGAG CAAGCGAAAA
Ppr_3          TGTGGGATCC TTTGAAGTGG TATATTCATT CATGGGGTTG GAGGTTAGAG GTATTTTGAG CAAGCGAAAA
Ppr_4          TGTGGGATCC TTTGAAGTGG TATATTCATT CATGGGGTTG GAGGTTAGAG GTATTTTGAG CAAGCGAAAA
Ppr_5          TGTGGGATCC TTTGAAGTGG TATATTCATT CATGGGGTTG GAGGTTAGAG GTATTTTGAG CAAGCGAAAA

               ....|....| ....|....| ....|....| ....|....| ....|....| ....|....| ....|....|
                       640        650        660        670        680        690        700
Peu_1          GGAGTGATTT TCAAGTGTGT CAGAGATGGG GTTGTGAGTG GAGAGAAATG GACTGTTCTT GTTGAAGAAG
Peu_2          GGAGTGATTT TCAAGTGTGT CAGAGATGGG GTTGTGAGTG GAGAGAAATG GACTGTTCTT GTTGAAGAAG
Peu_3          GGAGTGATTT TCAAGTGTGT CAGAGATGGG GTTGTGAGTG GAGAGAAATG GACTGTTCTT GTTGAAGAAG
Peu_4          GGAGTGATTT TCAAGTGTGT CAGAGATGGG GTTGTGAGTG GAGAGAAATG GACTGTTCTT GTTGAAGAAG
Peu_5          GGAGTGATTT TCAAGTGTGT CAGAGATGGG GTTGTGAGTG GAGAGAAATG GACTGTTCTT GTTGAAGAAG
Ppr_1          GGAGTGGTTT TCAAGTGTGT CAGAGATGGG GTTGYGAGTG GAGAGAAATG GACTGTTCTT GTTGAAGAAG
Ppr_2          GGAGTGGTTT TCAAGTGTGT CAGAGATGGG GTTGYGAGTG GAGAGAAATG GACTGTTCTT GTTGAAGAAG
Ppr_3          GGAGTGGTTT TCAAGTGTGT CAGAGATGGG GTTGYGAGTG GAGAGAAATG GACTGTTCTT GTTGAAGAAG
Ppr_4          GGAGTGGTTT TCAAGTGTGT CAGAGATGGG GTTGYGAGTG GAGAGAAATG GACTGTTCTT GTTGAAGAAG
Ppr_5          GGAGTGGTTT TCAAGTGTGT CAGAGATGGG GTTGYGAGTG GAGAGAAATG GACTGTTCTT GTTGAAGAAG

               ....|....| ....|....| ....|....| ....|....| ....|....| ....|....| ....|....|
                       710        720        730        740        750        760        770
Peu_1          AAGGAGGAGA ATTGAAGGTT GTTTCAAGGT TCAAGGCACG ACCATCCATT GGTGAAGTAG AGAATGTTTT
Peu_2          AAGGAGGAGA ATTGAAGGTT GTTTCAAGGT TCAAGGCACG ACCATCCATT GGTGAAGTAG AGAATGTTTT
Peu_3          AAGGAGGAGA ATTGAAGGTT GTTTCAAGGT TCAAGGCACG ACCATCCATT GGTGAAGTAG AGAATGTTTT
Peu_4          AAGGAGGAGA ATTGAAGGTT GTTTCAAGGT TCAAGGCACG ACCATCCATT GGTGAAGTAG AGAATGTTTT
```

```
Peu_5          AAGGAGGAGA ATTGAAGGTT GTTTCAAGGT TCAAGGCACG ACCATCCATT GGTGAAGTAG AGAATGTTTT
Ppr_1          AAGGAGGACA ATTGAAGGTT GTTTCAAGGT TCAAGGCACG ACCATCCATT GGTGAAGTAG AGAATGTTTT
Ppr_2          AAGGAGGACA ATTGAAGGTT GTTTCAAGGT TCAAGGCACG ACCATCCATT GGTGAAGTAG AGAATGTTTT
Ppr_3          AAGGAGGACA ATTGAAGGTT GTTTCAAGGT TCAAGGCACG ACCATCCATT GGTGAAGTAG AGAATGTTTT
Ppr_4          AAGGAGGACA ATTGAAGGTT GTTTCAAGGT TCAAGGCACG ACCATCCATT GGTGAAGTAG AGAATGTTTT
Ppr_5          AAGGAGGACA ATTGAAGGTT GTTTCAAGGT TCAAGGCACG ACCATCCATT GGTGAAGTAG AGAATGTTTT

               ....|....| ....|....| ....|....| ....|....| ....|....| ....|....| ....|....|
                       780        790        800        810        820        830        840
Peu_1          GTACAATCTG ATGGCTATCA ATTCGCCAAT TACCAAGTCT GCTAGGTTTT TCAAGGATTT GGTGTCAAAT
Peu_2          GTACAATCTG ATGGCTATCA ATTCGCCAAT TACCAAGTCT GCTAGGTTTT TCAAGGATTT GGTGTCAAAT
Peu_3          GTACAATCTG ATGGCTATCA ATTCGCCAAT TACCAAGTCT GCTAGGTTTT TCAAGGATTT GGTGTCAAAT
Peu_4          GTACAATCTG ATGGCTATCA ATTCGCCAAT TACCAAGTCT GCTAGGTTTT TCAAGGATTT GGTGTCAAAT
Peu_5          GTACAATCTG ATGGCTATCA ATTCGCCAAT TACCAAGTCT GCTAGGTTTT TCAAGGATTT GGTGTCAAAT
Ppr_1          GTACAATCTG ATGGCTATCA ATTCGCCAAT TACCAAGTCT GCTAGGTTTT TCAAGGATTT GGTGTCAAAT
Ppr_2          GTACAATCTG ATGGCTATCA ATTCGCCAAT TACCAAGTCT GCTAGGTTTT TCAAGGATTT GGTGTCAAAT
Ppr_3          GTACAATCTG ATGGCTATCA ATTCGCCAAT TACCAAGTCT GCTAGGTTTT TCAAGGATTT GGTGTCAAAT
Ppr_4          GTACAATCTG ATGGCTATCA ATTCGCCAAT TACCAAGTCT GCTAGGTTTT TCAAGGATTT GGTGTCAAAT
Ppr_5          GTACAATCTG ATGGCTATCA ATTCGCCAAT TACCAAGTCT GCTAGGTTTT TCAAGGATTT GGTGTCAAAT

               ...

Peu_1          GTA
Peu_2          GTA
Peu_3          GTA
Peu_4          GTA
Peu_5          GTA
Ppr_1          GTA
Ppr_2          GTA
Ppr_3          GTA
Ppr_4          GTA
Ppr_5          GTA
```

Unigene39162_All (proline-rich family protein)

```
                ....|....| ....|....| ....|....| ....|....| ....|....| ....|....| ....|....|
                          10         20         30         40         50         60         70
Peu_1           AATTATSTAA TTTGTTTCAA ATTTTTAGGT GGATACGAGG AAAATGAAGA TGGATGTTAT TAGACCATGG
Peu_2           AATTATSTAA TTTGTTTCAA ATTTTTAGGT GGATACGAGG AAAATGAAGA TGGATGTTAT TAGACCATGG
Peu_3           AATTATSTAA TTTGTTTCAA ATTTTTAGGT GGATACGAGG AAAATGAAGA TGGATGTTAT TAGACCATGG
Peu_4           AATTATSTAA TTTGTTTCAA ATTTTTAGGT GGATACGAGG AAAATGAAGA TGGATGTTAT TAGACCATGG
Peu_5           AATTATSTAA TTTGTTTCAA ATTTTTAGGT GGATACGAGG AAAATGAAGA TGGATGTTAT TAGACCATGG
Ppr_1           AATTATCTAA TTTGTTTCAA ATTTTTAGGT GGATACGAGG AAAATGAAGA TGGATGTTAT TAGACCATGG
Ppr_2           AATTATCTAA TTTGTTTCAA ATTTTTAGGT GGATACGAGG AAAATGAAGA TGGATGTTAT TAGACCATGG
Ppr_3           AATTATCTAA TTTGTTTCAA ATTTTTAGGT GGATACGAGG AAAATGAAGA TGGATGTTAT TAGACCATGG
Ppr_4           AATTATCTAA TTTGTTTCAA ATTTTTAGGT GGATACGAGG AAAATGAAGA TGGATGTTAT TAGACCATGG
Ppr_5           AATTATCTAA TTTGTTTCAA ATTTTTAGGT GGATACGAGG AAAATGAAGA TGGATGTTAT TAGACCATGG

                ....|....| ....|....| ....|....| ....|....| ....|....| ....|....| ....|....|
                          80         90        100        110        120        130        140
Peu_1           ATTGCTACTA GAGTGACGGA GCTTCTCGGA TTTGAAGATG AAGTTCTTAT CAACTTCATT TATGGCCTGC
Peu_2           ATTGCTACTA GAGTGACGGA GCTTCTCGGA TTTGAAGATG AAGTTCTTAT CAACTTCATT TATGGCCTGC
Peu_3           ATTGCTACTA GAGTGACGGA GCTTCTCGGA TTTGAAGATG AAGTTCTTAT CAACTTCATT TATGGCCTGC
Peu_4           ATTGCTACTA GAGTGACGGA GCTTCTCGGA TTTGAAGATG AAGTTCTTAT CAACTTCATT TATGGCCTGC
Peu_5           ATTGCTACTA GAGTGACGGA GCTTCTCGGA TTTGAAGATG AAGTTCTTAT CAACTTCATT TATGGCCTGC
Ppr_1           ATTGCTACTA GAGTGACGGA GCTTCTCGGA TTTGAAGATG AAGTTCTTAT CAACTTCATT TATGGCCTGC
Ppr_2           ATTGCTACTA GAGTGACGGA GCTTCTCGGA TTTGAAGATG AAGTTCTTAT CAACTTCATT TATGGCCTGC
Ppr_3           ATTGCTACTA GAGTGACGGA GCTTCTCGGA TTTGAAGATG AAGTTCTTAT CAACTTCATT TATGGCCTGC
Ppr_4           ATTGCTACTA GAGTGACGGA GCTTCTCGGA TTTGAAGATG AAGTTCTTAT CAACTTCATT TATGGCCTGC
Ppr_5           ATTGCTACTA GAGTGACGGA GCTTCTCGGA TTTGAAGATG AAGTTCTTAT CAACTTCATT TATGGCCTGC

                ....|....| ....|....| ....|....| ....|....| ....|....| ....|....| ....|....|
                         150        160        170        180        190        200        210
Peu_1           TTGATGGAAA GGTCTTATTG ATTTTCTGCA ATTAACCTAA CTTTTTTTAG TGTCTGAAAA TAGTTCATGT
Peu_2           TTGATGGAAA GGTCTTATTG ATTTTCTGCA ATTAACCTAA CTTTTTTTAG TGTCTGAAAA TAGTTCATGT
Peu_3           TTGATGGAAA GGTCTTATTG ATTTTCTGCA ATTAACCTAA CTTTTTTTAG TGTCTGAAAA TAGTTCATGT
Peu_4           TTGATGGAAA GGTCTTATTG ATTTTCTGCA ATTAACCTAA CTTTTTTTAG TGTCTGAAAA TAGTTCATGT
Peu_5           TTGATGGAAA GGTCTTATTG ATTTTCTGCA ATTAACCTAA CTTTTTTTAG TGTCTGAAAA TAGTTCATGT
Ppr_1           TTGATGGAAA GGTCTTATTG ATTTTCTGCA ATTAACCTAA CTTTTTTTAG TGTCTGAAAA TAGTTCATGT
Ppr_2           TTGATGGAAA GGTCTTATTG ATTTTCTGCA ATTAACCTAA CTTTTTTTAG TGTCTGAAAA TAGTTCATGT
Ppr_3           TTGATGGAAA GGTCTTATTG ATTTTCTGCA ATTAACCTAA CTTTTTTTAG TGTCTGAAAA TAGTTCATGT
Ppr_4           TTGATGGAAA GGTCTTATTG ATTTTCTGCA ATTAACCTAA CTTTTTTTAG TGTCTGAAAA TAGTTCATGT
Ppr_5           TTGATGGAAA GGTCTTATTG ATTTTCTGCA ATTAACCTAA CTTTTTTTAG TGTCTGAAAA TAGTTCATGT

                ....|....| ....|....| ....|....| ....|....| ....|....| ....|....| ....|....|
                         220        230        240        250        260        270        280
Peu_1           TTTGCTTTTC AATTGAATAC TGCACGCGTG GATCCTCTGT CTTTGCTAAT ATAATTTTAC AATACTTGCC
Peu_2           TTTGCTTTTC AATTGAATAC TGCACGCGTG GATCCTCTGT CTTTGCTAAT ATAATTTTAC AATACTTGCC
Peu_3           TTTGCTTTTC AATTGAATAC TGCACGCGTG GATCCTCTGT CTTTGCTAAT ATAATTTTAC AATACTTGCC
Peu_4           TTTGCTTTTC AATTGAATAC TGCACGCGTG GATCCTCTGT CTTTGCTAAT ATAATTTTAC AATACTTGCC
Peu_5           TTTGCTTTTC AATTGAATAC TGCACGCGTG GATCCTCTGT CTTTGCTAAT ATAATTTTAC AATACTTGCC
Ppr_1           TTTGCTTTTC AATTGAATAC TGCACGCGTG GATCCTCTGT CTTTGCTAAT ATAATTTTAC AATACTTGCC
Ppr_2           TTTGCTTTTC AATTGAATAC TGCACGCGTG GATCCTCTGT CTTTGCTAAT ATAATTTTAC AATACTTGCC
Ppr_3           TTTGCTTTTC AATTGAATAC TGCACGCGTG GATCCTCTGT CTTTGCTAAT ATAATTTTAC AATACTTGCC
Ppr_4           TTTGCTTTTC AATTGAATAC TGCACGCGTG GATCCTCTGT CTTTGCTAAT ATAATTTTAC AATACTTGCC
Ppr_5           TTTGCTTTTC AATTGAATAC TGCACGCGTG GATCCTCTGT CTTTGCTAAT ATAATTTTAC AATACTTGCC

                ....|....| ....|....| ....|....| ....|....| ....|....| ....|....| ....|....|
                         290        300        310        320        330        340        350
Peu_1           TAAAGAATTG TTGAGAGTTC GTTTGGTGTT AGGCATTTTG TTTCTTAACA ATAGTCAAGT GGTTTGGTTA
Peu_2           TAAAGAATTG TTGAGAGTTC GTTTGGTGTT AGGCATTTTG TTTCTTAACA ATAGTCAAGT GGTTTGGTTA
Peu_3           TAAAGAATTG TTGAGAGTTC GTTTGGTGTT AGGCATTTTG TTTCTTAACA ATAGTCAAGT GGTTTGGTTA
Peu_4           TAAAGAATTG TTGAGAGTTC GTTTGGTGTT AGGCATTTTG TTTCTTAACA ATAGTCAAGT GGTTTGGTTA
Peu_5           TAAAGAATTG TTGAGAGTTC GTTTGGTGTT AGGCATTTTG TTTCTTAACA ATAGTCAAGT GGTTTGGTTA
Ppr_1           TAAAGAATTG TTGAGAGTTC GTTTGGTGTT AGGCATTTTG TTTCTTAACA ATAGTCAAGT GGTTTGGTTA
Ppr_2           TAAAGAATTG TTGAGAGTTC GTTTGGTGTT AGGCATTTTG TTTCTTAACA ATAGTCAAGT GGTTTGGTTA
Ppr_3           TAAAGAATTG TTGAGAGTTC GTTTGGTGTT AGGCATTTTG TTTCTTAACA ATAGTCAAGT GGTTTGGTTA
Ppr_4           TAAAGAATTG TTGAGAGTTC GTTTGGTGTT AGGCATTTTG TTTCTTAACA ATAGTCAAGT GGTTTGGTTA
```

```
Ppr_5          TAAAGAATTG TTGAGAGTTC GTTTGGTGTT AGGCATTTTG TTTCTTAACA ATAGTCAAGT GGTTTGGTTA

               ....|....| ....|....| ....|....| ....|....| ....|....| ....|....| ....|....|
                      360        370        380        390        400        410        420
Peu_1          TCAATTTTTT TTTGTTGTAT ACTGTATTGA TGCAGGAAGT GAACGGGAAG GAGGTTCAAA TATCGCTTAC
Peu_2          TCAATTTTTT TTTGTTGTAT ACTGTATTGA TGCAGGAAGT GAACGGGAAG GAGGTTCAAA TATCGCTTAC
Peu_3          TCAATTTTTT TTTGTTGTAT ACTGTATTGA TGCAGGAAGT GAACGGGAAG GAGGTTCAAA TATCGCTTAC
Peu_4          TCAATTTTTT TTTGTTGTAT ACTGTATTGA TGCAGGAAGT GAACGGGAAG GAGGTTCAAA TATCGCTTAC
Peu_5          TCAATTTTTT TTTGTTGTAT ACTGTATTGA TGCAGGAAGT GAACGGGAAG GAGGTTCAAA TATCGCTTAC
Ppr_1          TCAATTTTTT TTTGTTGTAT ACTGTATTGA TGCAGGAAGT GAACGGGAAG GAGGTTCAAA TATCGCTTAC
Ppr_2          TCAATTTTTT TTTGTTGTAT ACTGTATTGA TGCAGGAAGT GAACGGGAAG GAGGTTCAAA TATCGCTTAC
Ppr_3          TCAATTTTTT TTTGTTGTAT ACTGTATTGA TGCAGGAAGT GAACGGGAAG GAGGTTCAAA TATCGCTTAC
Ppr_4          TCAATTTTTT TTTGTTGTAT ACTGTATTGA TGCAGGAAGT GAACGGGAAG GAGGTTCAAA TATCGCTTAC
Ppr_5          TCAATTTTTT TTTGTTGTAT ACTGTATTGA TGCAGGAAGT GAACGGGAAG GAGGTTCAAA TATCGCTTAC

               ....|....| ....|....| ....|....| ....|....| ....|....| ....|....| ....|....|
                      430        440        450        460        470        480        490
Peu_1          GGGATTCATG GAGAAAAACA CTGGGAAGTT TATGAAAGAG CTTTGGACAC TTCTTCTTAG TGCGCAGAAG
Peu_2          GGGATTCATG GAGAAAAACA CTGGGAAGTT TATGAAAGAG CTTTGGACAC TTCTTCTTAG TGCGCAGAAG
Peu_3          GGGATTCATG GAGAAAAACA CTGGGAAGTT TATGAAAGAG CTTTGGACAC TTCTTCTTAG TGCGCAGAAG
Peu_4          GGGATTCATG GAGAAAAACA CTGGGAAGTT TATGAAAGAG CTTTGGACAC TTCTTCTTAG TGCGCAGAAG
Peu_5          GGGATTCATG GAGAAAAACA CTGGGAAGTT TATGAAAGAG CTTTGGACAC TTCTTCTTAG TGCGCAGAAG
Ppr_1          GGGATTCATG GAGAAAAACA CTGGGAAGTT TATGAAAGAG CTTTGGACAC TTCTTCTTAG TGCGCAGAAA
Ppr_2          GGGATTCATG GAGAAAAACA CTGGGAAGTT TATGAAAGAG CTTTGGACAC TTCTTCTTAG TGCGCAGAAA
Ppr_3          GGGATTCATG GAGAAAAACA CTGGGAAGTT TATGAAAGAG CTTTGGACAC TTCTTCTTAG TGCGCAGAAA
Ppr_4          GGGATTCATG GAGAAAAACA CTGGGAAGTT TATGAAAGAG CTTTGGACAC TTCTTCTTAG TGCGCAGAAA
Ppr_5          GGGATTCATG GAGAAAAACA CTGGGAAGTT TATGAAAGAG CTTTGGACAC TTCTTCTTAG TGCGCAGAAA

               ....|....| ....|....| ....|....| ....|.
                      500        510        520
Peu_1          AATGAAAGTG GTGTTCCTCA ACAGTTTTTG GATGCC
Peu_2          AATGAAAGTG GTGTTCCTCA ACAGTTTTTG GATGCC
Peu_3          AATGAAAGTG GTGTTCCTCA ACAGTTTTTG GATGCC
Peu_4          AATGAAAGTG GTGTTCCTCA ACAGTTTTTG GATGCC
Peu_5          AATGAAAGTG GTGTTCCTCA ACAGTTTTTG GATGCC
Ppr_1          AATGAAAGTG GTGTTCCTCA ACAGTTTTTG GATGCC
Ppr_2          AATGAAAGTG GTGTTCCTCA ACAGTTTTTG GATGCC
Ppr_3          AATGAAAGTG GTGTTCCTCA ACAGTTTTTG GATGCC
Ppr_4          AATGAAAGTG GTGTTCCTCA ACAGTTTTTG GATGCC
Ppr_5          AATGAAAGTG GTGTTCCTCA ACAGTTTTTG GATGCC
```
