# Supplementary material for: Rapidly Evolving Genes and Stress Adaptation of Two Desert Poplars, Populus euphratica and P. pruinosa
Source: PLoS One. 2013 Jun 11;8(6):e66370. doi: 10.1371/journal.pone.0066370 (PMC3679102; doi:10.1371/journal.pone.0066370)
Supplement: Table S1 — Output Statistics of the sequencing. (DOCX) [file pone.0066370.s008.docx]

**Table S1** Output Statistics of the sequencing

| Samples | Total Reads | Total Nucleotides (nt) * | Q20 percentage | N percentage | GC percentage |
| --- | --- | --- | --- | --- | --- |
| control-callus | 28 139 789 | 4 220 968 350 | 95.20% | 0.03% | 43.81% |
| salt-stressed callus | 28 438 370 | 4 265 755 500 | 95.77% | 0.01% | 44.40% |
| desert-grown trees | 28 726 355 | 5 170 743 900 | 95.48% | 0.00% | 44.75% |
| Total | 85 304 514 | 12 795 677 100 |  |  |  |

* Total Nucleotides = Total Reads x Read size
