# Supplementary material for: Rapidly Evolving Genes and Stress Adaptation of Two Desert Poplars, Populus euphratica and P. pruinosa
Source: PLoS One. 2013 Jun 11;8(6):e66370. doi: 10.1371/journal.pone.0066370 (PMC3679102; doi:10.1371/journal.pone.0066370)
Supplement: Table S2 — GO categories of the P. pruinosa all-unigenes based on Blast2GO. (DOCX) [file pone.0066370.s009.docx]

**Table S2 GO categories of the *P. pruinosa* all-unigenes based on** Blast2GO

| Ontology | Term | Number | Percentage |
| --- | --- | --- | --- |
| biological_process | metabolic process | 4636 | 0.400103564 |
| biological_process | cellular process | 4427 | 0.382066109 |
| biological_process | biological regulation | 1090 | 0.094070942 |
| biological_process | pigmentation | 1014 | 0.087511867 |
| biological_process | localization | 978 | 0.084404937 |
| biological_process | establishment of localization | 975 | 0.084146026 |
| biological_process | response to stimulus | 734 | 0.063346854 |
| biological_process | cellular component organization | 289 | 0.024941745 |
| biological_process | multicellular organismal process | 239 | 0.020626564 |
| biological_process | developmental process | 234 | 0.020195046 |
| biological_process | cellular component biogenesis | 190 | 0.016397687 |
| biological_process | multi-organism process | 141 | 0.01216881 |
| biological_process | anatomical structure formation | 123 | 0.010615345 |
| biological_process | reproduction | 72 | 0.00621386 |
| biological_process | reproductive process | 69 | 0.00595495 |
| biological_process | death | 22 | 0.00189868 |
| biological_process | growth | 21 | 0.001812376 |
| biological_process | immune system process | 14 | 0.001208251 |
| biological_process | biological adhesion | 2 | 0.000172607 |
| biological_process | cell killing | 1 | 8.63E-05 |
| biological_process | locomotion | 1 | 8.63E-05 |
| biological_process | rhythmic process | 0 | 0 |
| biological_process | viral reproduction | 0 | 0 |
| cellular_component | cell | 7362 | 0.635367222 |
| cellular_component | cell part | 7360 | 0.635194615 |
| cellular_component | organelle | 5399 | 0.465953223 |
| cellular_component | macromolecular complex | 1033 | 0.089151635 |
| cellular_component | organelle part | 908 | 0.078363683 |
| cellular_component | envelope | 194 | 0.016742902 |
| cellular_component | extracellular region | 158 | 0.013635971 |
| cellular_component | membrane-enclosed lumen | 141 | 0.01216881 |
| cellular_component | virion | 11 | 0.00094934 |
| cellular_component | virion part | 11 | 0.00094934 |
| cellular_component | extracellular region part | 5 | 0.000431518 |
| cellular_component | symplast | 0 | 0 |
| cellular_component | synapse | 0 | 0 |
| cellular_component | synapse part | 0 | 0 |
| molecular_function | binding | 4958 | 0.427893329 |
| molecular_function | catalytic | 4557 | 0.393285579 |
| molecular_function | transporter | 653 | 0.056356261 |
| molecular_function | transcription regulator | 389 | 0.033572107 |
| molecular_function | structural molecule | 376 | 0.03245016 |
| molecular_function | electron carrier | 243 | 0.020971779 |
| molecular_function | molecular transducer | 203 | 0.017519634 |
| molecular_function | translation regulator | 106 | 0.009148183 |
| molecular_function | antioxidant | 69 | 0.00595495 |
| molecular_function | enzyme regulator | 46 | 0.003969966 |
| molecular_function | nutrient reservoir | 10 | 0.000863036 |
| molecular_function | metallochaperone | 2 | 0.000172607 |
| molecular_function | protein tag | 2 | 0.000172607 |
| molecular_function | auxiliary transport protein | 0 | 0 |
| molecular_function | chemoattractant | 0 | 0 |
| molecular_function | chemorepellent | 0 | 0 |
| molecular_function | proteasome regulator | 0 | 0 |
