# Supplementary material for: Rapidly Evolving Genes and Stress Adaptation of Two Desert Poplars, Populus euphratica and P. pruinosa
Source: PLoS One. 2013 Jun 11;8(6):e66370. doi: 10.1371/journal.pone.0066370 (PMC3679102; doi:10.1371/journal.pone.0066370)
Supplement: Table S3 — Orthologous pairs and alignment summary. (DOCX) [file pone.0066370.s010.docx]

**Table S3** Orthologous pairs and alignment summary

|  | Number | Mean Length (bp) | Range (bp) |
| --- | --- | --- | --- |
| CDS |  |  |  |
| *P. euphratica* | 59 721 | 532 | 102-6 309 |
| *P. pruinosa* | 68 526 | 437 | 102-4 422 |
| Orthologs |  |  |  |
| *P. euphratica* & *P. pruinosa* | 2 859 | 1 079 | 573-3 555 |
