# Supplementary material for: Rapidly Evolving Genes and Stress Adaptation of Two Desert Poplars, Populus euphratica and P. pruinosa
Source: PLoS One. 2013 Jun 11;8(6):e66370. doi: 10.1371/journal.pone.0066370 (PMC3679102; doi:10.1371/journal.pone.0066370)
Supplement: Table S5 — Annotation information for the 84 ortholog pairs with Ka/Ks >1. (DOCX) [file pone.0066370.s012.docx]

**Table S5** Annotation information for the 84 ortholog pairs with *Ka/Ks* > 1. Hit accessions are from the UniProt database. NA indicates an annotation was not available for the ortholog pair.

Gene ontology: F, molecular function, P, biological process, C, cellular components

| Ka/Ks | Seq. description | Hit accession number | Gene ontology |
| --- | --- | --- | --- |
| 7.220 | DNA-binding protein DF1 | B9H7S0 | F: DNA binding; p: regulation of transcription |
| 4.909 | cotton fiber expressed protein 1 | B9N362 | NA |
| 2.904 | zinc finger (C3HC4-type RING finger) family protein | B9GIZ8 | F: zinc ion binding |
| 2.298 | ethylene-responsive protein -related | B9R7Y7 | NA |
| 2.238 | NAC domain protein, IPR003441 (NAC146 ) | B9MVT6 | P: regulation of transcription, DNA-dependent; F: DNA binding |
| 2.208 | AP2/ERF domain-containing transcription factor (ERF82) | B9I767 | P: transcription, DNA-dependent; F: DNA binding; F: sequence-specific DNA binding transcription factor activity; C: nucleus |
| 2.129 | NA | B9HAT5 | NA |
| 2.023 | transcription factor | A9PGH6 | NA |
| 1.994 | myb-like transcription factor | B9I997 | F: DNA binding |
| 1.976 | EMB2759 (embryo defective 2759) | A9PIB3 | NA |
| 1.926 | Cytochrome P450 (CYP82D2) | B9MVH7 | F: electron carrier activity; F: heme binding; F: monooxygenase activity; F: oxidoreductase activity, acting on paired donors, with incorporation or reduction of molecular oxygen |
| 1.889 | kelch repeat-containing F-box family protein | B9HG98 | NA |
| 1.836 | AP2/ERF domain-containing transcription factor (ERF81) | B9INK5 | P: transcription, DNA-dependent; F: DNA binding; F: sequence-specific DNA binding transcription factor activity; C: nucleus |
| 1.802 | RTM2 (RESTRICTED TEV MOVEMENT 2) | B9HVH3 | NA |
| 1.766 | tetratricopeptide repeat (TPR)-containing protein | B9GRB6 | NA |
| 1.733 | ACBP2 | B9IDN1 | P: lipid metabolic process; P: response to cold; P: response to lead ion; F: fatty-acyl-CoA binding; F: ead ion binding; F: phosphatidic acid binding; C: endoplasmic reticulum; C: plant-type cell wall; C: plasma membrane |
| 1.678 | pseudo response regulator | B9N2R7 | P: intracellular signal transduction; P: regulation of transcription, DNA-dependent; F: DNA binding; F: two-component response regulator activity; C: intracellular |
| 1.657 | NA | B9H1Y7 | NA |
| 1.649 | lipoxygenase LOX-2 | B9IB24 | P: oxylipin biosynthetic process; F: iron ion binding; F: lipoxygenase activity |
| 1.630 | Proteinase inhibitor I4, serpin | B9GYI2 | NA |
| 1.624 | ubiquitin family protein | B9N6Y0 | NA |
| 1.620 | emb1379 (embryo defective 1379) | B9MYR0 | NA |
| 1.614 | F-box family protein | B9IHD0 | NA |
| 1.589 | NA | B9GQC2 | NA |
| 1.579 | ACBP3 | B9GNR2 | F: fatty-acyl-CoA binding |
| 1.537 | contains similarity to myosin heavy chain | B9N420 | NA |
| 1.513 | NA | B9HST0 | NA |
| 1.512 | UDP-glucose:glucosyltransferase | B9HL29 | F: transferase activity, transferring hexosyl groups |
| 1.508 | dihydropterin pyrophosphokinase-dihydropteroate synthase | B9HIG0 | P: response to oxidative stress; P: tetrahydrofolate biosynthetic process; F: 2-amino-4-hydroxy-6-hydroxymethyldihydropteridine diphosphokinase activity; F: dihydropteroate synthase activity; C: cytosol |
| 1.462 | NA | B9RB52 | NA |
| 1.428 | RhoGAP; Wiscott-Aldrich syndrome, C-terminal | B9HBY8 | P: signal transduction; C:intracellular |
| 1.426 | NA | B9GKQ9 | NA |
| 1.398 | LisH domain-containing protein C1711.05 | B9HN37 | NA |
| 1.388 | BURP domain-containing protein | B9HRQ1 | NA |
| 1.387 | kelch repeat-containing F-box family protein | B9GS45 | NA |
| 1.386 | RAD23; damaged DNA bindin | B9GXM9 | P: nucleotide-excision repair; P: proteasomal ubiquitin-dependent protein catabolic process; F: damaged DNA binding; C: nucleus |
| 1.371 | NA | A9PJ82 | NA |
| 1.359 | DNA-binding protein PD1 | B9GKI4 | F: DNA binding |
| 1.355 | NGA1 (NGATHA1); transcription factor | B9RMW1 | P: regulation of transcription, DNA-dependent; P: transcription, DNA-dependent; F: DNA binding; C: nucleus |
| 1.333 | similar to cytidine/deoxycytidylate deaminase family protein | B9GQ87 | P: riboflavin biosynthetic process; F: diaminohydroxyphosphoribosylaminopyrimidine deaminase activity; F: oxidoreductase activity; F: zinc ion binding; C: chloroplast |
| 1.328 | Probable caffeoyl-CoA O-methyltransferase (CCoAOMT) | B9N5I7 | F: O-methyltransferase activity |
| 1.315 | zinc finger (C2H2 type) family protein | B9GSY9 | F: zinc ion binding; C: intracellular |
| 1.309 | NA | B9H2N7 | NA |
| 1.306 | eukaryotic initiation factor 4 | B9HB41 | P: RNA metabolic process; P: response to virus; P: spread of virus in host, cell to cell; F: DNA binding; F: RNA binding; C: cytoplasm; C: nucleus |
| 1.300 | ATSIK | B9SRS3 | F: protein kinase activity; F: ATP binding |
| 1.299 | SEU3A protein | B9S2M5 | F: transcription cofactor activity; C: nucleus |
| 1.298 | glycosyltransferase | B9GGB4 | F: cis-zeatin O-beta-D-glucosyltransferase activity; F: trans-zeatin O-beta-D-glucosyltransferase activity |
| 1.284 | reticulon family protein (RTNLB5) | B9GQE4 | C: endoplasmic reticulum |
| 1.268 | F-box family protein | B9IMC2 | NA |
| 1.257 | NHL repeat-containing protein | B9GUT4 | NA |
| 1.255 | armadillo/beta-catenin repeat family protein | B9H214 | F: binding |
| 1.246 | myb family transcription factor | B9HIZ7 | F: DNA binding; P: regulation of transcription; P: primary root development; P: cellular response to phosphate starvation; C: nucleus |
| 1.241 | WAK-like kinase | A9PJ27 | F: ATP binding; F: protein serine/threonine kinase activity |
| 1.220 | ECT10 | B9HBC4 | NA |
| 1.218 | ubiquitin fusion degradation protein | A9PDL8 | P: ubiquitin-dependent protein catabolic process |
| 1.218 | calcium-binding mitochondrial protein-related | B9HGI2 | F: calcium ion binding; C: mitochondrion |
| 1.202 | NA | B9I0C3 | NA |
| 1.185 | heat stress transcription factor A3 | B9SRG8 | F: sequence-specific DNA binding; F: sequence-specific DNA binding transcription factor activity; C: nucleus |
| 1.182 | NA | B9GP96 | C: chloroplast |
| 1.177 | with no lysine kinase | B9RTS8 | F: ATP binding; F: protein serine/threonine kinase activity |
| 1.160 | ATP-dependent Clp protease proteolytic subunit | B9H362 | P: proteolysis; F: serine-type endopeptidase activity; C: chloroplast envelope; C: chloroplast thylakoid; C: chloroplastic endopeptidase Clp complex |
| 1.160 | protein transport protein-related | B9ID81 | C: chloroplast |
| 1.146 | NA | B9H5G0 | NA |
| 1.141 | proteasome-related | A9PEG4 | P: protein folding; F: binding |
| 1.138 | VAP27-2 (VAMP/SYNAPTOBREVIN-ASSOCIATED PROTEIN 27-2) | D7TUW5 | F: structural molecule activity |
| 1.135 | pentatricopeptide (PPR) repeat-containing protein | B9N4R0 | NA |
| 1.127 | wax synthase isoform 3 | B9HJR2 | NA |
| 1.117 | UDP-glucoronosyl/UDP-glucosyl transferase family protein | B9GM96 | F: transferase activity, transferring hexosyl groups |
| 1.103 | NA | B9GKN6 | P: rRNA processing; C: nucleus |
| 1.097 | proline-rich family protein | B9SHS4 | NA |
| 1.095 | Cysteine protease ATG4; Autophagy-related protein 4 | B9N8L2 | P: autophagy; P: protein transport; P: proteolysis; F: cysteine-type peptidase activity; C: cytoplasm |
| 1.089 | NAC domain protein, IPR003441 (NAC036 ) | B9HZY3 | P: regulation of transcription, DNA-dependent; P: response to abscisic acid stimulus; P: response to water deprivation; F: DNA binding; C: nucleus |
| 1.070 | NADPH quinone oxidoreductase-like protein | B9H236 | F: nucleotide binding; F: oxidoreductase activity; F: zinc ion binding; |
| 1.054 | translation initiation factor-related | B9HE58 | NA |
| 1.049 | twin LOV protein 1 | B9HTE0 | P: regulation of transcription, DNA-dependent; P: signal transduction by phosphorylation; F: two-component sensor activity |
| 1.044 | NA | B9RGF0 | NA |
| 1.043 | PDLP2 (PLASMODESMATA-LOCATED PROTEIN 2) | B9HKU7 | P: plasmodesmata-mediated intercellular transport; C: plasmodesma |
| 1.040 | NA | A9PGU5 | P: proteolysis; F: aspartic-type endopeptidase activity |
| 1.040 | Ripening-related protein grip22 | B9STV8 | NA |
| 1.034 | 2OG-Fe(II) oxygenase | F6GWN3 | F: oxidoreductase activity, acting on paired donors, with incorporation or reduction of molecular oxygen, 2-oxoglutarate as one donor, and incorporation of one atom each of oxygen into both donors |
| 1.031 | extracellular matrix structural constituent | B9GQP6 | NA |
| 1.025 | mitochondrial substrate carrier family protein | B9IFZ1 | P: transmembrane transport; C: integral to membrane |
| 1.022 | Methyltransferase small | B9GMZ3 | F: methyltransferase activity; F: nucleic acid binding |
| 1.013 | plastid DNA-binding protein | B9HUW7 | NA |
